# Self-reinoculation with fecal flora changes microbiota density and composition leading to an altered bile-acid profile in the mouse small intestine

**DOI:** 10.1186/s40168-020-0785-4

**Published:** 2020-02-12

**Authors:** Said R. Bogatyrev, Justin C. Rolando, Rustem F. Ismagilov

**Affiliations:** 1 Division of Biology and Biological Engineering, California Institute of Technology, Pasadena, CA USA; 2 Division of Chemistry and Chemical Engineering, California Institute of Technology, 1200 E. California Blvd, Pasadena, CA USA

**Keywords:** Microbial quantification, Metabolomics analyses, Mouse models, Small intestine microbiota, Bile acids, Deconjugation, Coprophagy, Microbial colonization, 16S rRNA gene amplicon sequencing

## Abstract

**Background:**

The upper gastrointestinal tract plays a prominent role in human physiology as the primary site for enzymatic digestion and nutrient absorption, immune sampling, and drug uptake. Alterations to the small intestine microbiome have been implicated in various human diseases, such as non-alcoholic steatohepatitis and inflammatory bowel conditions. Yet, the physiological and functional roles of the small intestine microbiota in humans remain poorly characterized because of the complexities associated with its sampling. Rodent models are used extensively in microbiome research and enable the spatial, temporal, compositional, and functional interrogation of the gastrointestinal microbiota and its effects on the host physiology and disease phenotype. Classical, culture-based studies have documented that fecal microbial self-reinoculation (via coprophagy) affects the composition and abundance of microbes in the murine proximal gastrointestinal tract. This pervasive self-reinoculation behavior could be a particularly relevant study factor when investigating small intestine microbiota. Modern microbiome studies either do not take self-reinoculation into account, or assume that approaches such as single housing mice or housing on wire mesh floors eliminate it. These assumptions have not been rigorously tested with modern tools. Here, we used quantitative 16S rRNA gene amplicon sequencing, quantitative microbial functional gene content inference, and metabolomic analyses of bile acids to evaluate the effects of self-reinoculation on microbial loads, composition, and function in the murine upper gastrointestinal tract.

**Results:**

In coprophagic mice, continuous self-exposure to the fecal flora had substantial quantitative and qualitative effects on the upper gastrointestinal microbiome. These differences in microbial abundance and community composition were associated with an altered profile of the small intestine bile acid pool, and, importantly, could not be inferred from analyzing large intestine or stool samples. Overall, the patterns observed in the small intestine of non-coprophagic mice (reduced total microbial load, low abundance of anaerobic microbiota, and bile acids predominantly in the conjugated form) resemble those typically seen in the human small intestine.

**Conclusions:**

Future studies need to take self-reinoculation into account when using mouse models to evaluate gastrointestinal microbial colonization and function in relation to xenobiotic transformation and pharmacokinetics or in the context of physiological states and diseases linked to small intestine microbiome and to small intestine dysbiosis.

Video abstract.

## Background

The small intestine is the primary site for enzymatic digestion and nutrient uptake, immune sampling, and drug absorption in the human gastrointestinal system. Its large surface area vastly exceeds that of the large intestine [1], and thus may serve as a broad interface for host-microbial interactions.

A growing body of scientific evidence highlights the importance of the small intestine microbiome in normal human physiology and response to dietary interventions [2, 3]. Alterations in the small intestine microbiome are implicated in a number of human disorders, such as malnutrition [4, 5], obesity, and metabolic disease [6], inflammatory bowel disease (IBD) and irritable bowel syndrome (IBS) [7–9], and drug side effects [10]. Despite the apparent importance of the small intestine microbiome in human health, it remains understudied and poorly characterized largely because of the procedural and logistical complexities associated with its sampling in humans (methods are too invasive and require specialized healthcare facilities). Moreover, microbial composition tends to differ substantially among the small intestine, large intestine, and stool of the same animal or human subject [11, 12], which highlights the importance of targeted sampling of the small intestine for analyses.

Mice are the predominant animal species of model organisms in the field of microbiome research. Compared with other mammalian models, mice have a lower cost of maintenance, their environment and diet can be easily controlled, they are amenable to genetic manipulation, there are numerous genetic mouse models already available, and propagation using inbred colonies reduces inter-individual variability [13]. Additionally, murine germ-free (GF) and gnotobiotic technologies are well established. Using mouse models enables interrogation of the entire gastrointestinal tract (GIT) and examination of the changes in microbiome and host physiology that occur in response to experimental conditions (e.g., dietary modifications, xenobiotic administration) or microbial colonization (e.g., monocolonization, colonization with defined microbial consortia, human microbiota-associated mice).

Rodent models also have several well-recognized limitations associated with their genetic, anatomical, and physiological differences with humans [13, 14]. Among these limitations is the persistent tendency of rodents to practice gastrointestinal auto- and allo-reinoculation with large intestine microbiota (via fecal ingestion, or coprophagy) in laboratory settings [15–17]. This pervasive behavior has been documented in classical studies using observational techniques in both conventional and GF mice [18], in conventional mice maintained on standard and fortified diets [19], in animals with and without access to food [20], and across different mouse strains [16, 21].

Multiple classical studies have attempted to evaluate the effects of self-reinoculation on the structure of the microbiota in the rodent small intestine [22–24] and large intestine and stool [20, 23, 25, 26] using traditional microbiological techniques, but reported conflicting results [23, 25, 26]. This lack of consensus may be attributed to the use of different methods for preventing coprophagy (some of which are ineffective), non-standardized diets, inter-strain or inter-species differences among the animal models, or other unaccounted for experimental parameters. It has been also suggested that repeated self-exposure in mice via coprophagy can promote microbial colonization of the GIT by “exogenous” microbial species, such as *Pseudomonas* spp. [27]. All of these observations highlight the importance of considering self-reinoculation in studies of gastrointestinal microbial ecology in murine models. However, the field currently lacks precise and comprehensive evaluations of the effects of self-reinoculation on the spatial, structural, and functional state of the gut microbiome and its effects on murine host physiology. Current microbiome studies in rodents either do not take self-reinoculation into account, or assume it can be eliminated by single housing of animals or housing them on wire mesh floors (also referred to as “wire screens” or “wire grids”) [14]. Despite classical literature suggesting these assumptions can be incorrect [16, 21, 28–32], they have not been tested on mice housed in modern facilities using state-of-the-art quantitative tools.

Here, we explicitly test these assumptions about murine self-reinoculation to answer the following three questions relevant to gastrointestinal microbiome research: (1) Do quantitative 16S rRNA gene amplicon sequencing tools detect differences in small intestine microbial loads between mice known to be coprophagic and non-coprophagic? (2) Does coprophagy impact the microbial composition of the small intestine? (3) Do differences in microbiota density and composition associated with self-reinoculation in mice impact microbial function (e.g., alter microbial metabolite production or modifications) in the small intestine?

To answer these questions, we analyzed gastrointestinal samples from mice under conditions known to prevent coprophagy (fitting with “tail” or “fecal collection” cups [16, 23, 26, 30, 33]) and typical laboratory conditions in which mice are known to be coprophagic (housing in standard cages). We also included samples from single-housed mice in standard and wire-floor cages. We analyzed the quantitative and compositional changes in the microbiome along the entire length of the mouse GIT in response to self-reinoculation, computationally inferred the changes in microbial function, and evaluated the microbial function-related metabolite profiles in the corresponding segments of the gut.

## Results

We first performed a pilot study to confirm that preventing coprophagy in mice would result in decreased viable microbial load and altered microbiota composition in the small intestine. We used a most probable number (MPN) assay utilizing anaerobic BHI-S broth medium to evaluate the live (culturable) microbial loads along the entire GIT of mice known to be coprophagic (housed in standard cages in groups, *N* = 5) and mice known to be non-coprophagic (fitted with tail cups and housed in standard cages in groups, *N* = 5). Consistent with the published classical literature [20, 24], we found that coprophagic mice had significantly higher loads of culturable microbes in their upper GIT than mice that were non-coprophagic (Additional file 1: Figure S4A). Moreover, the microbial community composition in the proximal GIT, particularly in the stomach, of coprophagic mice more closely resembled the microbial composition of the large intestine (Additional file 1: Figure S4B) as revealed by 16S rRNA gene amplicon sequencing (*N* = 1 mouse analyzed from each group) and principal component analysis (PCA) of the resulting relative abundance data.

**Fig. 1:**
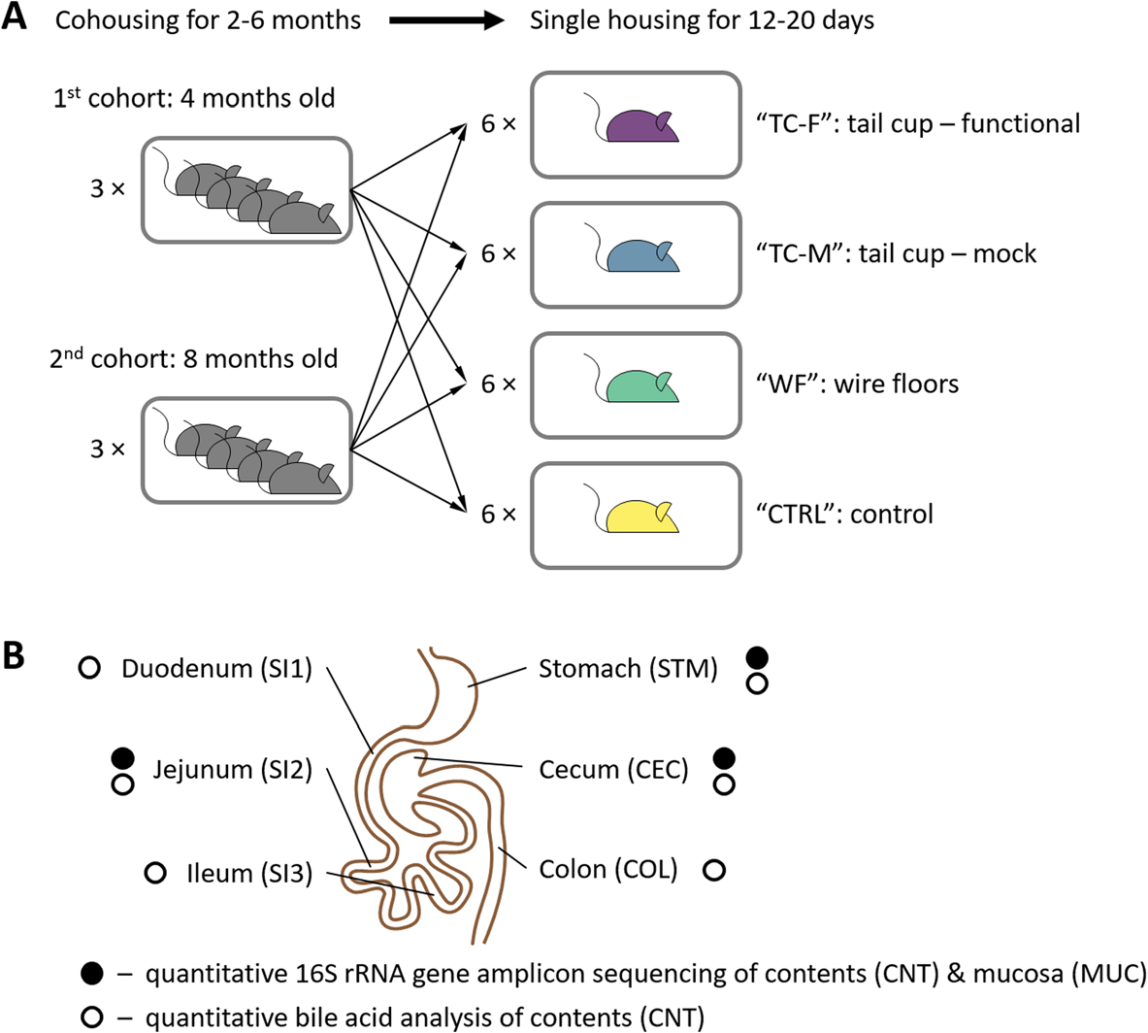
An overview of the study design and timeline. **a** Mice from two age cohorts (4-month-old and 8-month-old) were raised co-housed (four mice to a cage) for 2–6 months. One mouse from each cage was then assigned to one of the four experimental conditions: functional tail cups (TC-F), mock tail cups (TC-M), housing on wire floors (WF), and controls housed in standard conditions (CTRL). All mice were singly housed and maintained on each treatment for 12–20 days (*N* = 24, 6 mice per group). **b** Samples were taken from six sites throughout the gastrointestinal tract. Each sample was analyzed by quantitative 16S rRNA gene amplicon sequencing of lumenal contents (CNT) and mucosa (MUC) and/or quantitative bile-acid analyses of CNT. Panel **b** is adapted from [13, 34])

This pilot study confirmed that in our hands, tail cups were effective at preventing the self-reinoculation of viable fecal flora in the upper GIT of mice. These results spurred us to design a rigorous, detailed study (Fig. 1) to answer the three questions posed above using state-of-the-art methods: quantitative 16S rRNA gene amplicon sequencing (to account for both changes in the total microbial load and the unculturable taxa), quantitative functional gene content inference, and targeted bile-acid metabolomics analyses.

The study design (Fig. 1) consisted of six cages of four animals each that were co-housed for 2–6 months and then split into four experimental groups and singly housed for 12–20 days. The four experimental conditions were the following: animals fitted with functional tail cups (TC-F) and singly housed in standard cages, animals fitted with mock tail cups (TC-M) and singly housed in standard cages, animals singly housed on wire floors (WF), and control animals singly housed in standard conditions (CTRL). At the end of the study, gastrointestinal contents and mucosal samples were collected from all segments of the GIT of each animal and we evaluated total microbial loads (entire GIT) and microbiome composition (stomach (STM), jejunum (SI2), and cecum (CEC)).

We chose the cecum segment of the large intestine for quantitative 16S rRNA gene amplicon sequencing because the analysis of the contents of this section can provide a complete snapshot of the large intestine and fecal microbial diversity in response to environmental factors [35–37]. Cecal contents also enabled us to collect a more consistent amount of sample from all animals across all experimental conditions (whereas defecation may be inconsistent among animals at the time of terminal sampling).

### Self-reinoculation increases microbial loads in the upper gut

**Fig. 2:**
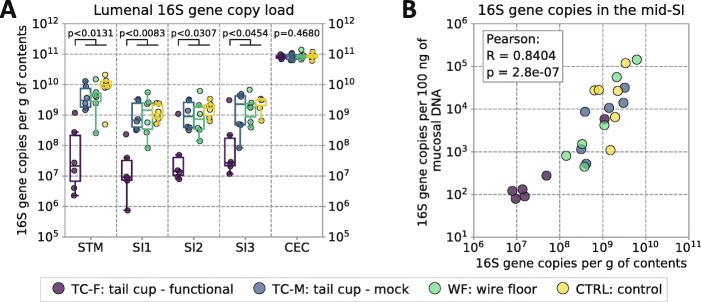
Quantification of microbial loads in lumenal contents and mucosa of the gastrointestinal tracts (GIT) of mice in the four experimental conditions: functional tail cups (TC-F), mock tail cups (TC-M), housing on wire floors (WF), and controls housed in standard conditions (CTRL). **a** Total 16S rRNA gene DNA copy loads, a proxy for total microbial loads, were measured along the GIT of mice of all groups (STM = stomach; SI1 = upper third of the small intestine (SI), SI2 = middle third of the SI, SI3 = lower third of the SI roughly corresponding to the duodenum, jejunum, and ileum respectively; CEC = cecum; COL = colon). Multiple comparisons were performed using a Kruskal–Wallis test, followed by pairwise comparisons using the Wilcoxon–Mann–Whitney test with false-discovery rate (FDR) correction. Individual data points are overlaid onto box-and-whisker plots; whiskers extend from the quartiles (Q2 and Q3) to the last data point within 1.5 × interquartile range (IQR). **b** Correlation between the microbial loads in the lumenal contents (per gram total contents) and in the mucosa (per 100 ng of mucosal DNA) of the mid-SI. *N* = 6 mice per experimental group

To answer our first question (Can quantitative sequencing tools detect the difference in 16S rRNA gene DNA copy load in the upper GIT of mice known to be coprophagic and non-coprophagic?), we analyzed total quantifiable microbial loads across the GIT using 16S rRNA gene DNA quantitative PCR (qPCR) and digital PCR (dPCR). Preventing self-reinoculation in mice equipped with functional tail cups dramatically decreased the lumenal microbial loads in the upper GIT but not in the lower GIT (Fig. 2a). Total quantifiable microbial loads in the upper GIT were reduced only in mice equipped with functional tail cups. All other experimental groups of singly-housed animals (those equipped with mock tail cups, housed on wire floors, or housed on standard woodchip bedding) that retained access to fecal matter and practiced self-reinoculation had similarly high microbial loads in the upper GIT, as expected from the published literature [16, 21, 28–32].

Across all test groups, mucosal microbial loads in the mid-small intestine demonstrated high correlation (Pearson’s *R* = 0.84, *P* = 2.8 × 10^− 7^) with the microbial loads in the lumenal contents (Fig. 2b).

Stomach (STM) and small intestine (SI1, SI2, and SI3) samples from one (out of six) of the TC-F mice showed higher microbial loads compared with the other TC-F mice. The total microbial load in the upper GIT in this TC-F mouse was similar to mice from all other groups (TC-M, WF, CTRL), which emphasizes the crucial importance of performing analyses of both microbial load and composition (discussed below) on the same samples.

### Self-reinoculation substantially alters the microbiota composition in the upper gut but has less pronounced effects in the large intestine

**Fig. 3:**
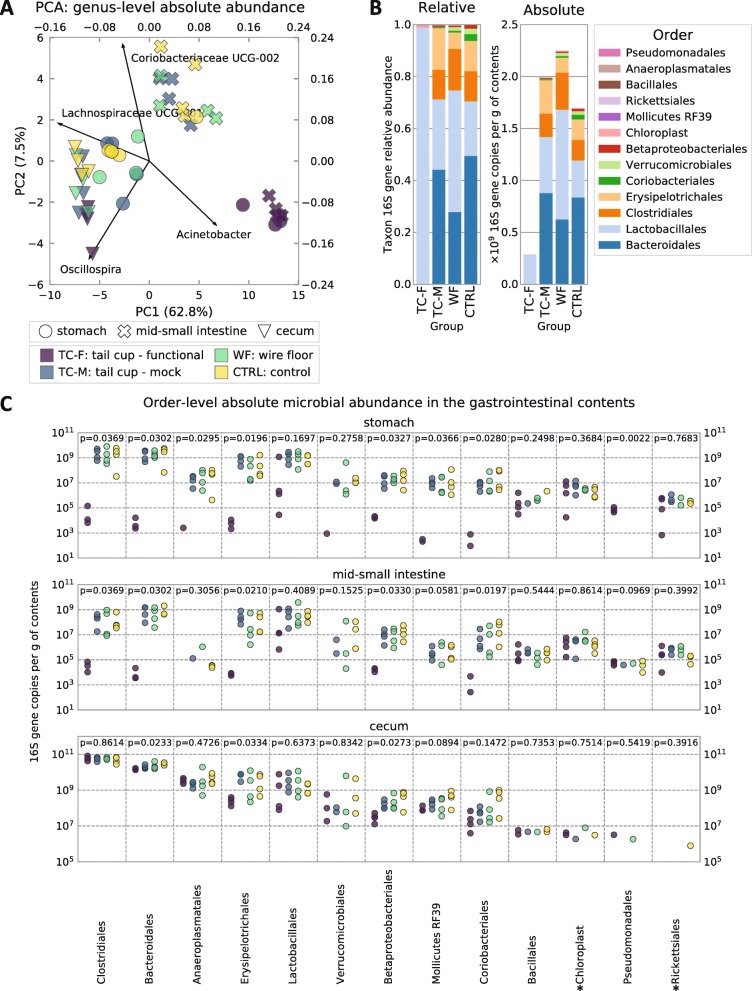
Compositional and quantitative 16S rRNA gene amplicon sequencing analysis of the gut microbiota. **a** Principal component analysis (PCA) of the log_10_-transformed and standardized (mean = 0, SD = 1) absolute microbial abundance profiles in the stomach, mid-small intestine, and cecum. Loadings of the top contributing taxa are shown for each principal component. **b** Mean relative and absolute abundance profiles of microbiota in the mid-SI (order level) for all experimental conditions. Functional tail cups (TC-F), mock tail cups (TC-M), housing on wire floors (WF), and controls housed in standard conditions (CTRL). N = 6 mice per experimental group, 4 of which were used for sequencing. **c** Absolute abundances of microbial taxa (order level) compared between coprophagic and non-coprophagic mice along the mouse GIT. *Chloroplast and *Richettsiales (mitochondria) represent 16S rRNA gene DNA amplicons from food components of plant origin. Multiple comparisons were performed using the Kruskal–Wallis test

To answer our second question (Does self-reinoculation with fecal microbiota impact upper GIT microbial composition?), we performed quantitative 16S rRNA gene amplicon sequencing [38, 39] (Barlow JT, Bogatyrev SR, Ismagilov RF: A quantitative sequencing framework for absolute abundance measurements of mucosal and lumenal microbial communities, submitted) on the stomach (STM), jejunum (SI2), and cecum (CEC) samples. Qualitative sequencing revealed dramatic overall changes in the upper GIT microbiota caused by self-reinoculation (Fig. 3). An exploratory PCA performed on the multidimensional absolute microbial abundance profiles highlights the unique and distinct composition of the upper GIT microbiome of non-coprophagic mice (Fig. 3a). It is noteworthy that the stomach (STM) and small intestine (SI2) microbiota in all coprophagic mice clustered closer to the large intestine microbiota, suggesting the similarity was due to persistent self-reinoculation with the large intestine microbiota (Fig. 3a).

Self-reinoculation had differential effects across microbial taxa (Fig. 3c), which could be classified into three main categories depending on the pattern of their change as follows:

1. “Fecal taxa” (e.g., *Clostridiales*, *Bacteroidales, Erysipelotrichales*) that either dropped significantly or disappeared (fell below the lower limit of detection [LLOD] of the quantitative sequencing method [38] (Barlow JT, Bogatyrev SR, Ismagilov RF: A quantitative sequencing framework for absolute abundance measurements of mucosal and lumenal microbial communities, submitted)) in the upper GIT of non-coprophagic mice;
2. “True small intestine taxa” (e.g., *Lactobacillales*) that remained relatively stable in the upper GIT in non-coprophagic mice;
3. Taxa that had lower absolute abundance in the cecum (e.g., *Bacteroidales*, *Erysipelotrichales*, *Betaproteobacteriales*) of non-coprophagic (compared with coprophagic) mice.

Overall, the composition of the small intestine microbiota of coprophagic mice was consistent with that previously reported in literature [35]. The upper GIT microbiota in non-coprophagic mice was dominated by *Lactobacilli* (Fig. 3c), known to be a prominent microbial taxon in human small intestine microbiota [3, 40, 41]. Importantly, the compositional analysis showed that the single TC-F mouse that had high microbial loads in its stomach and small intestine had a microbial composition in those segments of the GIT similar (i.e., dominated by *Lactobacillales*) to all other TC-F mice, and very distinct from all coprophagic mice (Fig. 3b, c). The PCA showed that the stomach and mid-small intestine of this mouse clustered with the stomach and mid-small intestine of all other TC-F mice (Fig. 3a).

### Changes in the small intestine microbiota lead to differences in inferred microbial functional gene content

We hypothesized that the quantitative and qualitative changes in the small intestine microbiota induced by self-reinoculation may result in altered microbial function [42, 43] and an altered metabolite profile, either indirectly, as a result of functional changes in the microbiota, or directly via re-ingestion of fecal metabolites. To understand how such alterations to microbiota would impact microbial function in the small intestine, we next aimed to predict how the absolute abundances of functional microbial genes would be affected. We coupled the pipeline for microbial functional inference based on the 16S rRNA marker gene sequences (PICRUSt2) [44, 45] with our quantitative 16S rRNA gene amplicon sequencing approach [38] (Barlow JT, Bogatyrev SR, Ismagilov RF: A quantitative sequencing framework for absolute abundance measurements of mucosal and lumenal microbial communities, submitted). We focused our analysis on microbial functions that would be highly relevant to small intestine physiology: microbial conversion of host-derived bile acids and microbial modification of xenobiotics.

**Fig. 4:**
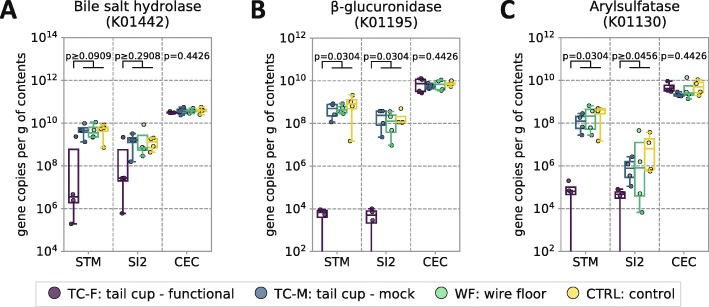
Inference of microbial genes involved in bile-acid and xenobiotic conjugate modification along the GIT of coprophagic and non-coprophagic mice. Inferred absolute abundance of the microbial genes encoding (**a**) bile salt hydrolases (cholylglycine hydrolases), (**b**) beta-glucuronidases, and (**c**) arylsulfatases throughout the GIT (*STM* stomach, *SI2* middle third of the small intestine (SI) roughly corresponding to the jejunum, *CEC* cecum). KEGG orthology numbers are given in parentheses for each enzyme. In all plots, individual data points are overlaid onto box-and-whisker plots; whiskers extend from the quartiles (Q2 and Q3) to the last data point within 1.5 × interquartile range (IQR). Multiple comparisons were performed using the Kruskal–Wallis test; pairwise comparisons were performed using the Wilcoxon–Mann–Whitney test with FDR correction. *N* = 4 mice per group

We found that the inferred absolute abundances of a number of microbial gene orthologs implicated in enzymatic hydrolysis of conjugated bile acids (bile salt hydrolase, BSH [46–48]) and xenobiotic conjugates (e.g., beta-glucuronidase, arylsulfatase [49, 50]) in the stomach and the small intestine of coprophagic mice were dramatically higher (in some cases by several orders of magnitude) than in non-coprophagic mice (Fig. 4). This difference was not observed in the cecum.

### Changes in the small intestine microbiota induced by self-reinoculation alter the bile acid profile

Bile acids are a prominent class of host-derived compounds with multiple important physiological functions and effects on the host and its gut microbiota [51, 52]. These host-derived molecules are highly amenable to microbial modification in both the small and large intestine [53]. The main microbial bile acid modifications in the GIT include deconjugation, dehydrogenation, dehydroxylation, and epimerization [52]. Thus, we next performed quantitative bile acid profiling along the entire GIT to evaluate the effects of self-reinoculation on bile acid composition.

The small intestine is the segment of the GIT that harbors the highest levels of bile acids (up to 10 mM) and where they function in lipid emulsification and absorption [54–56]. Given these high concentrations of bile acid substrates, we specifically wished to analyze whether the differences we observed in small intestine microbiota (Figs. 2 and 3) between coprophagic and non-coprophagic mice would result in pronounced effects on microbial deconjugation of bile acids. We also wished to test whether any differences in bile acid deconjugation were in agreement with the differences in the absolute BSH gene content we inferred (Fig. 4a) from the absolute microbial abundances (Fig. 3c).

**Fig. 5:**
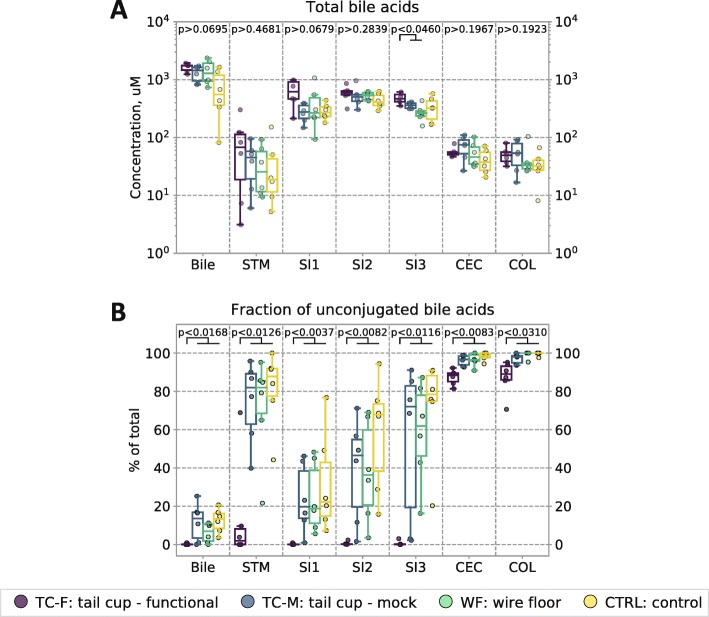
Bile acid profiles in gallbladder bile and in lumenal contents along the entire GIT. **a** Total bile acid levels (conjugated and unconjugated; primary and secondary) and **b** the fraction of unconjugated bile acids in gallbladder bile and throughout the GIT (*STM* stomach; *SI1* upper third of the small intestine (SI), *SI2* middle third of the SI, *SI3* lower third of the SI roughly corresponding to the duodenum, jejunum, and ileum respectively; *CEC* cecum; *COL* colon). In all plots, individual data points are overlaid onto box-and-whisker plots; whiskers extend from the quartiles (Q2 and Q3) to the last data point within 1.5 × interquartile range (IQR). Multiple comparisons were performed using the Kruskal–Wallis test; pairwise comparisons were performed using the Wilcoxon–Mann–Whitney test with FDR correction. *N* = 6 mice per group

We first confirmed that in all four experimental groups, total bile acids levels (conjugated and unconjugated; primary and secondary) across all sections of the GIT were highest in the small intestine (Fig. 5a). We then compared the levels of conjugated and unconjugated (Fig. 5b) as well as primary (host-synthesized) and secondary (microbe-modified) bile acids (Additional file 1: Figure S5) between coprophagic and non-coprophagic mice.

Across all sections of the GIT and in the bile, non-coprophagic mice (TC-F) had significantly lower levels of unconjugated bile acids compared with coprophagic mice (Fig. 5b). Consistent with the computational inference in Fig. 4a (performed on mid-SI samples only), in all three sections of the small intestine of non-coprophagic mice (TC-F), the levels of unconjugated bile acids were substantially lower than in coprophagic mice. Almost 100% of the total bile acid pool remained in a conjugated form in the small intestine of non-coprophagic mice.

In all groups of coprophagic mice (TC-M, WF, and CTRL) the fraction of unconjugated bile acids gradually increased from the proximal to distal end of the small intestine. Gallbladder bile acid profiling (Fig. 5b) confirmed that bile acids were secreted into the duodenum predominantly in the conjugated form in all coprophagic mice. This pattern is consistent with the hypothesis that the exposure of bile acids to microbial deconjugation activity increases as they transit down a small intestine with high microbial loads (Fig. 2a) [54].

In the large intestine, non-coprophagic (TC-F) mice carried a smaller fraction of unconjugated bile acids compared with all coprophagic experimental groups (Fig. 5b).

Bile acid deconjugation in the small intestine of coprophagic mice was uniform for all glyco- and tauro-conjugates of all primary and secondary bile acids measured in our study (Additional file 1: Table S7), suggesting a broad-specificity BSH activity was provided by a complex fecal flora in the small intestine of those animals.

In the gallbladder bile and across all segments of the GIT from the stomach to the cecum, non-coprophagic mice had a statistically significantly lower fraction (but not lower absolute levels) of total secondary bile acids (conjugated and unconjugated) than coprophagic mice (Additional file 1: Figure S5). This change was uniform for the entire secondary bile acid pool of those analyzed (Additional file 1: Table S7). The only segment of the gut in which the difference in the fraction of secondary bile acids was not statistically significant between coprophagic and non-coprophagic mice was the colon. In fact, the differences in the fractions of total unconjugated and total secondary bile acids between coprophagic and non-coprophagic mice would have gone largely undetected had we only analyzed colonic contents or stool. These findings further highlight the importance of the comprehensive spatial interrogation of the complex crosstalk between the microbiota and bile acids in the gastrointestinal tract.

## Discussion

In this study, we used modern tools for quantitative microbiota profiling and showed that when self-reinoculation with fecal flora is prevented, the mouse small intestine harbors dramatically lower densities of microbiota and an altered microbial profile. Consistent with published literature [16, 21, 28–32], we confirmed that single housing on wire floors failed to prevent mice from practicing coprophagy and that only functional tail cups reliably prevented the self-reinoculation with fecal flora.

Despite its effectiveness, the tail cup approach has limitations. Tail cups in their current design may not be suitable for female rodents due to anatomical differences leading to urine entering and remaining inside the devices [57]. Animals need to be singly housed to prevent them from gnawing on each other’s tail cups and causing device failure or injury. The tail cup approach may be hard to implement in younger and actively growing mice (e.g., before or around weaning). Some mice in our study developed self-inflicted skin lesions from over-grooming at the location where the tail cups come in contact with the body at the animal’s hind end. Thus, we concluded that the approach in its current implementation is limited to 2–3 weeks in adult animals.

Our device design reduced the risk of tail injury and necrosis described in previous works [33] and allows for emptying the cups only once every 24 h to reduce handling stress. Because host stress can affect the microbiota [58] and other physiological parameters, we included a mock tail cup group. Both TC-F and TC-M mice demonstrated a similar degree of weight loss (Additional file 1: Figure S3A) when compared with the WF and CTRL mice despite similar food intake rates across all four groups (Additional file 1: Figure S3B). Mice fitted with mock tail cups (TC-M) had microbial patterns and bile acid profiles similar to the CTRL mice, thus the effects on the upper GIT microbiota and bile acid profiles that we observed in non-coprophagic (TC-F) mice are not attributable to stress.

We believe that the tail cup approach is implementable in gnotobiotic settings (e.g., flexible film isolators and individually ventilated cages), which can aid studies that involve association of mice with defined microbial communities or with human-derived microbiota.

### The non-coprophagic mouse model may be more relevant to humans

Using quantitative microbiota profiling, our study demonstrated that preventing self-reinoculation dramatically reduced the total levels of several prominent taxonomical groups of obligate anaerobes (e.g., *Clostridiales*, *Bacteroidales*, *Erysipelotrichale*) in the upper gastrointestinal microbiota of conventional mice. Despite these differences in taxa, levels of *Lactobacillales* in the small intestine and cecum, but not in the stomach, remained similar between coprophagic and non-coprophagic animals (Fig. 3c). The physiological significance of the maintained persistent population of *Lactobacillales* in the upper gastrointestinal tract (e.g., stomach or small intestine) and their overall consistent presence along the entire GIT [14, 59] for the host is not fully understood. However, *Lactobacilli* colonization in the stomach and small intestine has been shown to promote resistance to colonization by pathogens (reviewed in [60, 61]).

Compared with conventional (coprophagic) mice, the non-coprophagic mice displayed features of the small intestine microbiota and bile acid profiles that are more similar to the patterns seen in the small intestine of humans: orders of magnitude lower microbiota density, reduced abundance of obligate anaerobic flora and dominance of *Lactobacillales*, and a higher ratio of conjugated bile acids. These findings highlight the need to understand and control self-reinoculation in mouse models used to answer questions relevant to host-microbiota interactions in human health.

### Self-reinoculation and microbial ecology in the mouse GIT

We observed that within the approximately 2-week timeframe of our study, the taxonomical diversity of the mouse large intestine microbiome was stable in the absence of persistent microbial self-reinoculation: all taxonomical groups at the order level observed in the cecum of coprophagic mice were present in the cecum of non-coprophagic mice, and vice versa.

The trending changes in the absolute abundances of several taxa (*Bacterodales, Erysipelotrichales*, and *Betaproteobacteriales*) in the large intestine of non-coprophagic mice may be the result of eliminated self-reinoculation and/or the consequence of the altered profile of bile acids entering the cecum from the small intestine, or other undetected changes in the biochemical environment. Additionally, changes in the absolute abundance of some taxa may lead to changes in the absolute abundances of other metabolically coupled taxa. It has been previously suggested that the degree of bile acid deconjugation may alter the microbiota profile [46]. *Erysipelotrichales* (including *Turicibacter* spp*.*) in the mouse ileum and cecum have been shown to be positively correlated with unconjugated ileal and cecal [62] and plasma [63] bile acids. *Bacteroidales* (including *Muribaculum* spp*.*) in the cecum increased upon dietary supplementation of unconjugated cholic and chenodeoxycholic acids [64] and *Betaproteobacteriales* (including *Parasutterella* spp.) were positively correlated with unconjugated primary and secondary bile acids [65, 66] in mice. Thus, the decrease in the fraction of unconjugated bile acids in the large intestine of non-coprophagic mice (Fig. 5b) may be responsible for the decreased absolute abundance of these three taxonomic groups in the cecum of non-coprophagic mice. It is of note that most of the published reports describe correlations between bile acid profiles and microbiota composition based on relative abundance data and without accounting for the inherent compositionality of relative abundance data [67], which is known to introduce inaccuracies in the correlation analysis [64, 68]. For improved correlation analysis, our study reports absolute abundances of the taxa, which could lead to discrepancies between such correlations observed in this study and previously published studies.

Stability of complex microbiomes in response to perturbations with and without continuous species reintroduction is an important subject of research in microbial ecology [69, 70]. Eliminating fecal ingestion provides a way to study stability and recovery of the mouse gut microbiota (e.g., in response to dietary change or antibiotic exposure [71]) in a way more relevant to modern humans. Thus, the non-coprophagic mouse model can significantly aid such research.

### Self-reinoculation with fecal flora leads to altered bile acid profiles in the GIT

We demonstrated that changes to small intestine microbiota density and composition had pronounced effects on microbial function resulting in increased bile acid deconjugation in that segment of the GIT. Bile acid deconjugation is a microbiota-mediated process that in healthy humans is conventionally believed to take place in the distal small intestine (ileum) and in the large intestine [72] such that sufficient lipid emulsification (with conjugated bile acids) and absorption can take place in the small intestine by the time digesta reaches the ileum [73]. As a result of the much higher bile acid concentrations in the small intestine compared with the large intestine, altered deconjugation of bile acids in the small intestine may have more wide-ranging effects on the entire enterohepatic system. Our data indicate that bile acid deconjugation can take place in any segment of the small intestine of conventional healthy mice as a function of the microbial density and composition (Figs. 2a, 3 and 5b), which is consistent with previous findings in animal models and in humans with small intestinal bacterial overgrowth (SIBO) [74–78].

Strikingly, the very low degree of bile acid deconjugation in the small intestine of non-coprophagic mice in our study resembles profiles seen in germ-free animals [79–81], gnotobiotic animals colonized only with microbes incapable of deconjugating bile acids [82–85], and antibiotic-treated animals [86–88]. Our observations suggest a mechanistic link between the small intestine microbiota density and composition and the bile acid modification in this segment of the GIT. The small intestine of healthy human subjects is believed to harbor bile acids predominantly in the conjugated form [89], which further substantiates that (compared with coprophagic mice) the small intestine of non-coprophagic mice is more similar to the small intestine of a healthy human.

Although microbiota density and composition in the large intestine of coprophagic and non-coprophagic mice were largely similar, non-coprophagic mice had a higher fraction of bile acids that remained in the conjugated form in the large intestine (Fig. 4b), likely as a result of the bile acids entering the large intestine from the ileum predominantly in a conjugated form. Additionally, across all study groups, the total concentrations of bile acids in the small intestine were ~ 10-fold greater than in the large intestine. We therefore infer that in coprophagic mice, a greater absolute amount of bile acids underwent deconjugation in the small intestine than in the large intestine; i.e., in coprophagic mice, the small intestine contaminated with high loads of fecal flora was the primary site of bile acid deconjugation.

Regulation of bile acid deconjugation activity in the gut is considered a potential health-promoting modality in a number of contexts, including lowering blood cholesterol levels (reviewed in [90–92]). BSH-active probiotics can be a promising delivery vehicle for promoting increased bile acid deconjugation in the gut. Our study emphasizes the importance of controlling for self-reinoculation when using mice to study the effects of BSH-active microbial strains or probiotics [48, 93–98] (especially those with high selectivity for particular bile acid conjugates [47, 82, 85]) because conventional (coprophagic) mice already have pronounced BSH activity in their small intestines. A non-coprophagic mouse may be a better animal model in such studies.

Our findings also have implications for the use of conventional (coprophagic) mice in diet studies. Deconjugated bile acids are less effective than conjugated bile acids at lipid emulsification and fat micelle formation [74, 99]. Increased bile acid deconjugation in the small intestine of animals and humans can lead to lipid malabsorption and fat-soluble vitamin deficiency, and in extreme scenarios even to steatorrhea [77, 100]. Previous research has shown that the small intestine microbiota plays an important role in mediating the effect of high-fat diets on the host [101]; our results suggest that future studies of the microbiota-mediated effects of high-fat diets need to consider increased microbial bile acid deconjugation in the mouse intestine due to self-reinoculation with fecal flora.

Bile acid deconjugation is considered to be obligatory [84, 102, 103] before the secondary bile acid metabolism (believed to be predominantly occurring in the large intestine [72]) can take place. These reactions in many cases are carried out by different members of the microbiota. Thus, the reduction of the deconjugation activity in the small intestine of non-coprophagic mice and consequently lower availability of free primary bile acids for further microbial modification can explain the decrease in the secondary bile acid fraction (percentage of all bile acids) in the bile acid pool across the GIT and gallbladder bile of non-coprophagic mice in our study. A similar but more pronounced trend has been observed in rabbits [104]. Reduced oral intake and recycling of fecal secondary bile acids as a result of eliminating coprophagy may also be a contributing factor to the lower fraction of secondary bile acids in the total bile acid pool in the enterohepatic circulation in these animals.

Total bile acid levels in the stomach were similar in coprophagic and non-coprophagic mice (and agree with literature [104, 105]); however, bile acid profiles (including the fraction of total unconjugated and total secondary bile acids) were substantially different. Surprisingly, in all coprophagic mice the fraction of unconjugated bile acids in the stomach appeared to be intermediate between the profiles in the small intestine and in the large intestine (Fig. 5b), suggesting that the bile acids in the stomach of coprophagic mice could be accumulating from bile acids re-ingested in feces and bile acids refluxed from the duodenum. This pattern was not observed in non-coprophagic mice, suggesting that coprophagy may alter the bile acid profile in the upper GIT both directly (via re-ingestion of fecal metabolites) and indirectly (via altered microbiota function).

### Inferences about microbial function in bile acid and drug modification

Our quantitative functional gene inference analysis predicted differential absolute abundance of the BSH orthologs between the small intestine of coprophagic and non-coprophagic mice (Fig. 4a). This approach has limitations associated with incomplete gene annotations, limited ability to infer metagenomes from the marker gene sequences when multiple microbial strains with similar 16S rRNA gene sequences exist [44, 45], difficulty to predict the exact gene expression and enzyme activity and specificity. To test our prediction about BSH we employed a targeted bile acid metabolomic analysis of mouse gastrointestinal samples and observed the differences in the small intestine bile acid deconjugation between coprophagic and non-coprophagic mice (Fig. 5b) that were in agreement with the differences in the inferred BSH gene abundances in the small intestine of those two types of animals (Fig. 4a). Interestingly, despite similar inferred BSH gene abundance in the cecum of coprophagic and non-coprophagic mice, the fraction of unconjugated bile acids in the cecum and colon of non-coprophagic mice was statistically significantly lower (Fig. 5b) compared with coprophagic mice.

Michaelis–Menten constants (*K*_*m*_) for many known BSH isoforms are in the range of hundreds of nanomoles [72]—similar to the levels of bile acids observed in the small intestine of all groups of mice in this study (Fig. 5a). Total bile acid levels in the cecum and colon were ~ 10-fold lower than those in the small intestine, and thus they were ~ 10-fold lower than the BSH *K*_*m*_ (Fig. 5a). The predominantly conjugated form of the bile acids arriving into the cecum from the small intestine of non-coprophagic mice and their absolute concentration (~ 10-fold lower than BSH *K*_*m*_) can potentially explain the lower degree of bile acid deconjugation in the large intestine of these animals (compared with coprophagic mice) on the timescale of normal gastrointestinal transit.

This highlights the importance of considering functional inference (based on either taxonomy or in silico hidden state prediction [44, 45, 106, 107]) in the context of a variable biochemical environment (e.g., substrate availability) and the host gastrointestinal physiology (secretion, gastrointestinal transit, absorption and transport, etc.) and warrants functional validation (e.g., metabolomics). Additionally, the validity of functional inference based on 16S rRNA gene sequence counts (from next generation sequencing) versus absolute 16S rRNA gene sequence abundances (this study) should be further explored in future work.

We next explored the effects of self-reinoculation on the absolute abundance of microbial gene orthologs implicated in xenobiotic modification [108] in the small intestine, as microbiota-dependent drug modification and toxicity in the small intestine have been previously observed in rodents [109–119]. Many drugs administered to humans and mice both via enteral and parenteral routes after reaching the systemic circulation are transformed by the liver into conjugates (e.g., glucuronic acid, sulphate, or glutathione conjugates) and excreted with bile into the GIT lumen. Such transformations are believed to reduce the small intestine reabsorption of xenobiotics and promote their excretion from the body with stool. Alterations in the small intestine microbiota may also lead to increased hydrolysis of such conjugates by microbial enzymes and promote the local toxicity of the drug and enable its re-uptake from the small intestine (i.e., undergo enterohepatic circulation) [10, 116], resulting in an increase in the xenobiotic flux through the liver [120, 121] and to an overall microbiota-dependent change in drug pharmacokinetics.

As with the inferred differential BSH absolute abundances (correlating activity of which we confirmed with the bile acid deconjugation measurements), our analysis predicted differences in the absolute abundance (Fig. 4b, c) of the microbial gene orthologs responsible for drug conjugate hydrolysis (e.g., beta-glucuronidases, sulfohydrolases) between the small intestine of coprophagic and non-coprophagic mice. If this prediction is further experimentally confirmed, it would imply that self-reinoculation must be controlled for or taken into account when investigating drug pharmacology in mice.

### Relevance of self-reinoculation in probiotics research

Many studies on probiotics and their effects on host animal physiology rely on repeated oral administration of live probiotic microorganisms to rodents. Our study suggests that self-reinoculation with live fecal flora in laboratory mice could both interfere with and introduce inconsistencies in live probiotic administration regimens. As has been stated earlier, particular attention should be given to self-reinoculation and its effects on the small intestine bile acid profile in studies aiming to evaluate the health effects of probiotics and other therapeutic modalities [48, 90–98] targeting bile acid deconjugation and metabolism.

### Relevance of mouse models in human microbiota research

The role of mouse models in human microbiota research remains a subject of debate [13, 14, 122]. At the same time, the field is recognizing the importance of reproducibility in gut microbiota research that uses mouse models [58, 122]. Several recent studies have highlighted the variability in lab-mouse microbiota related to animal strains and sources of origin [36, 123–127]. Others have attempted to catalog a “normal” or “core” gut microbiome [128, 129] and its spatial organization [35, 36] and function [130] in laboratory and wild mice. Recently, the small intestine microbiome has become the focus of studies conducted in mice in the context of host physiology [101] and disease [4, 131]. Yet, little attention has been given to the impact of self-reinoculation on the gut microbiota spatial structure and function, or to how study outcomes might be affected by controlling (or not controlling) for this experimental parameter in mouse models.

Self-reinoculation in rodents may affect not only their native microbiota, but also individual microbial colonizers [24] (e.g., in gnotobiotic animals) and complex xenomicrobiota (e.g., in human microbiota-associated (HMA) mice). HMA mice have emerged as an important research model for dissecting the mechanistic connection between the gut microbiota and the host phenotype in health and disease, even though the field acknowledges its limitations [132, 133]. Compositional differences between the small intestine and large intestine microbiomes in primates and humans [12, 40, 41] appear to be more substantial than those reported for laboratory mice [35, 130]. Our study emphasizes that the compositional similarity between small and large intestine microbiota in conventional laboratory mice can be a result of self-reinoculation with fecal flora. Thus, the effects of self-reinoculation on the spatial organization and function of human microbiota in HMA mice warrant future exploration.

## Conclusions

In conclusion, this study uses modern tools to demonstrate the importance of self-reinoculation in the context of microbial ecology and function within the mammalian gastrointestinal system. Our work highlights the importance of recognizing and properly controlling for self-reinoculation when murine studies analyzing small intestine microbiota, and its function intend to draw parallels with human physiology and pathophysiology. Additionally, spatial interrogation of the gut microbiota and its function in mouse models is important because even dramatic changes in the small intestine microbiome profile, function, and metabolome may be overlooked if only large intestine and stool samples are analyzed.

## Methods

### Experimental animals

All animal handling and procedures were performed in accordance with the California Institute of Technology (Caltech) Institutional Animal Care and Use Committee (IACUC). C57BL/6 J male specific-pathogen-free (SPF) mice were obtained at the age of 7–8 weeks from Jackson Laboratory (Sacramento, CA, USA) and housed four mice per cage. Two cohorts of animals were used: the first cohort was allowed to acclimate in the Caltech animal facility for 2 months and mice were 4 months old at the start of the study; the second cohort acclimated for 6 months and mice were 8 months old at the start of the study.

All animals were maintained on chow diet (PicoLab Rodent Diet 20 5053, LabDiet, St. Louis, MO, USA) and autoclaved water ad lib and subjected to a daily 13:11 light:dark cycle during acclimation and throughout the entire study. Mice were given measured amounts of food, and food intake during the experiment was measured by weighing the food during weekly cage changes and at the end time point for each animal. Body weight was measured at the start of the experiment, during weekly cage changes, and at the end time point.

### Animal housing conditions

During the experiment, all mice were singly housed in autoclaved cages (Super Mouse 750, Lab Products, Seaford, DE, USA). The mice in the control (CTRL), mock tail cup (TC-M), and functional tail cup (TC-F) treatments were housed on heat-treated hardwood chip bedding (Aspen Chip Bedding, Northeastern Products, Warrensburg, NY, USA) and provided with tissue paper (Kleenex, Kimberly-Clark, Irving, TX, USA) nesting material. The mice in the WF treatment were housed on raised wire floors with a mesh size of 3 × 3 per square inch (#75016, Lab Products) and provided with floorless paper huts (#91291, Shepherd Specialty Papers, Watertown, TN, USA). A thin layer of woodchip bedding was added under the wire floors to absorb liquid waste from the animals (Additional file 1: Figure S1D).

### Tail cup design and mounting

We designed the tail cups based on published literature [30, 134–137], including the locking mechanism [30]. Each cup was locked in place around the hind end of animals by anchoring to a tail sleeve designed with a perpendicular groove. Such tail sleeves allow for the cup to be held snugly against the animal so that the total weight of the tail cup is distributed along a large surface area of the tail skin, which minimizes complications. When mounted, the tail cups can freely rotate along the longitudinal axis, which ensures the locking mechanism does not strangulate the tail.

We hand-made the tail cups from 20 mL syringes (#4200.000 V0 Norm-Ject 20 mL Luer-Lock, Henke-Sass Wolf GmbH, Tuttlingen, Germany) as depicted on Additional file 1: Figure S1A-C. Multiple perforations were designed to accelerate desiccation of the captured fecal pellets. Lateral slits allowed for increasing the diameter of the locking edge; pressing on the slits with two fingers allowed tail cups to be quickly unfastened from tail sleeves. Mock tail cups were modified with wide gaps in the walls to allow the fecal pellets to fall out of the cup.

To prevent mice from gnawing on the plastic parts of the tail cups (which could create a jagged edge and lead to a subsequent injury), they were reinforced with metal flared rings made from stainless steel grommets (#72890, SS-4, C.S. Osborne, Harrison, NJ, USA) that were modified to reduce their size and weight. Metal rings were attached to tail cups using 4-mm-wide rubber rings cut from latex tubing (Amber Latex Rubber Tubing #62996–688, 1/2″ ID, 3/4″ OD; VWR, Radnor, PA, USA).

Tail sleeves were made from high-purity silicone tubing (HelixMark 60–411–51, 1/8“ ID, 1/4” OD; Helix Medical, Carpinteria, CA, USA). The tubing was split longitudinally, and a 2.0-mm-wide longitudinal strip of the wall was removed to accommodate for variable tail diameters among animals, and along the length of the tail to prevent uneven tail compression, and to facilitate uniform application of the tissue adhesive. The perpendicular tail cup mounting groove was made using a rotary tool (Craftsman #572.610530, Stanley Black & Decker, New Britain, CT, USA) equipped with a cutting disc (RD1, Perma-Grit Tools, Lincolnshire, UK). Each tail cup and sleeve together weighed approximately 4.12 g empty.

Before mounting the tail cups, animals were anesthetized for 10 min with isoflurane and placed on a heating pad to maintain body temperature. Sleeves were de-greased on the inside using 70% ethanol, and a veterinary tissue adhesive (GLUture Topical Adhesive #32046, Abbott Laboratories, Lake Bluff, IL, USA) was applied to the tail base. The adhesive was allowed to cure for 5 min, and then tail cups were mounted. Mice were returned back to their cages and allowed to recover from the anesthesia and ambulate.

Tail cups were emptied of fecal pellets daily at 08:00 AM. Mice were prompted to enter a restrainer [138] made from a black polypropylene 50-mL conical tube (TB5000 LiteSafe, Cole-Parmer, Vernon Hills, IL, USA), and the tail cups were unclipped and quickly emptied. Any residue on the tail cup was cleaned using a paper towel and Rescue solution (Virox Technologies, Oakville, ON, Canada) prior to the cups being remounted. Animals fitted with the mock tail cups were subjected to the identical procedure to match the handling conditions.

Tail cups were mounted on animals for a duration of between 12 and 20 days. All TC-F animals were time-matched with TC-M animals, (i.e., each animal from the TC-F group had a time-matched animal from the TC-M group handled and euthanized at the same time).

### Sample collection and treatment

All mice were euthanized as approved by the Caltech IACUC in accordance with the American Veterinary Medical Association Guidelines on Euthanasia [139]. Mice were euthanized while under isoflurane anesthesia (delivered via a calibrated gas vaporizer in an induction chamber followed by maintenance on a nose cone) via cardiac puncture followed by cervical dislocation. Blood was collected using a 1-mL syringe (#309659, Becton Dickinson) and 21G × 1″ needle (#26414, EXELINT International, Redondo Beach, CA, USA).

Blood was immediately placed into K_2_EDTA plasma separation tubes (MiniCollect 450480, Greiner Bio-One GmbH, Kremsmünster, Austria), gently mixed, and stored on ice for up to 1 h prior to centrifugation. Bile and urine were collected directly from the gall and urinary bladders respectively using a 1-mL syringe (#4010.200 V0 Norm-Ject 1-mL Tuberculin Luer, Henke-Sass Wolf GmbH) and 27G × 1/2″ needle (#26400, EXELINT International) and stored on ice.

Fecal samples were collected if present at the time of euthanasia. The entire gastrointestinal tract was excised from the gastroesophageal junction to the anal sphincter and stored on ice during processing.

#### Plasma separation

Blood samples were centrifuged in the plasma separation tubes at 2000 RCF for 5 min at 4 °C. Plasma was separated and stored at − 80 °C.

#### Processing of GIT contents

To prepare samples for the main experimental analyses (Figs. 2, 3, and 4), each mouse GIT was split into stomach, three-equal-length thirds of the small intestine, cecum, and colon. Contents from each segment of the GIT were flushed out using 2–5 mL of cold (4 °C) sterile autoclaved saline solution (0.9% NaCl (#S5886, Sigma-Aldrich) in ultrapure water (Milli-Q, MilliporeSigma, Burlington, MA, USA) followed by very gentle squeezing with tweezers to avoid mucosal damage. All samples were stored on ice during processing.

An aliquot of each sample diluted in saline was concentrated by centrifugation at 25,000 RCF for 10 min at 4 °C. The supernatant was removed and the pellet was reconstituted in 9 volumes of 1× DNA/RNA Shield (DRS) solution (R1100-250, Zymo Research, Irvine, CA, USA), mixed by vortexing and stored at – 80 °C for future DNA extraction. Separate aliquots of each sample were stored at − 80 °C for the metabolomic (bile acid) analysis.

Preparation of GIT contents for the MPN-based microbial quantification and 16S rRNA gene amplicon sequencing (pilot study; Additional file 1: Figure S4B) was the same as above, but conducted inside a vinyl anaerobic chamber (Coy Laboratory Products, Grass Lake, MI, USA) in an atmosphere of 5% hydrogen, 10% carbon dioxide, and 85% nitrogen. All samples were maintained on ice and immediately processed for the culture-based assay.

#### Preparation of GIT mucosa

After flushing its contents, each segment of the GIT was gently rinsed in sterile cold (~ 4 °C) saline, cut longitudinally, and placed flat on a glass slide. The mucosa was scraped from the tissue gently using a second clean glass slide. Glass slides (VistaVision #16004-422, VWR) were sterilized by dry heat sterilization at 200 °C for at least 2 h. Mucosal scrapings were collected and combined with 9 volumes of DRS solution, mixed by vortexing, and stored at − 80 °C in preparation for DNA and RNA extraction.

### Most probable number (MPN) assay

For the pilot study (Additional file 1: Figure S4A), the MPN assays (adapted from [140–144]) were performed on each GIT section (stomach, three sub-sections of the small intestine, cecum, and colon) from five mice fitted with functional tail cups and five control mice. The growth medium was brain heart infusion broth (Bacto BHI, #237500, Becton Dickinson, Franklin Lakes, NJ, USA), prepared in ultrapure water (Milli-Q), sterilized by autoclaving, allowed to cool to room temperature, and supplemented with 1.0 mg/L vitamin K_1_ (#L10575, Alfa Aesar, Haverhill, MA, USA), 5 mg/L hematin (#H3281, Sigma-Aldrich St. Louis, MO, USA), and 0.25 g/L l-cysteine (#168149, Sigma-Aldrich). The medium was allowed to equilibrate inside the anaerobic chamber for at least 24 h before use.

MPN assays were performed in clear, sterile, non-treated polystyrene 384-well plates (Nunc 265202, Thermo Fisher Scientific, Waltham, MA, USA). Two series of eight consecutive 10-fold serial dilutions were prepared from each sample in sterile autoclaved saline solution (equilibrated inside the anaerobic chamber for at least 24 h) on clear sterile non-treated polystyrene 96-well plates (Corning Costar 3370, Corning, NY, USA). We injected 10 μL of each serial dilution from each series into four (eight total per dilution) culture-medium replicates (wells) filled with 90 μL of the BHI-S broth medium.

Plates were sealed with a breathable membrane (Breath-Easy BEM-1, Diversified Biotech) and incubated for 5 days at 37.0 °C inside the anaerobic chamber. The plates were lidless for the first 24 h to facilitate uniform gas equilibration, then from 24 h to the end of the incubation period (120 h), a plastic lid was kept over the plates.

At the end of the incubation, the plates were scanned using a flatbed scanner (HP ScanJet 8250, Hewlett-Packard, Palo Alto, CA, USA) in the reflective mode with black background at 300 dpi resolution. The positive wells (replicates) were called by visually observing each acquired high-resolution image. The MPN for each sample was calculated using Microsoft Excel with the “Calc_MPN” macro [145].

### DNA extraction

DNA was extracted from thawed GIT contents and mucosal sample aliquots preserved in DRS solution with the ZymoBIOMICS DNA Miniprep Kit (D4300, Zymo Research) according to the manufacturer’s instructions. Samples were homogenized on a bead beater (MiniBeadBeater-16, Model 607, Bio Spec Products, Bartlesville, OK, USA) for 5 min at the default speed of 3450 RPM. Quantitative recovery of DNA across multiple orders of microbial loads in the samples was previously verified in [38, 39].

DNA yield and purity in the extracts was evaluated via light absorbance (NanoDrop 2000c, Thermo Fisher Scientific) and via a fluorometric assay (Qubit dsDNA HS Assay Kit Q32854, Thermo Fisher Scientific) on a fluorometer (Invitrogen Qubit 3, Thermo Fisher Scientific).

### Quantitative PCR (qPCR) for 16S rRNA gene DNA copy enumeration

The qPCR reactions were set up in triplicates for each DNA sample. A single replicate reaction volume of 15 μL contained 1.5 μL of the DNA extracts combined with the qPCR master mix (SsoFast EvaGreen Supermix, #172-5200, Bio-Rad Laboratories, Hercules, CA, USA), forward and reverse primers (synthesized by Integrated DNA Technologies, San Diego, CA, USA; Additional file 1: Table S1) at a final concentration of 500 nM, and ultrapure water (Invitrogen UltraPure DNase/RNase-Free Distilled Water 10977-015, Thermo Fisher Scientific). Reactions were set up in white 96-well PCR plates (#HSP9655, Bio-Rad Laboratories) sealed with a PCR tape (#MSB1001, Bio-Rad Laboratories).

The standard curve was built for each qPCR run based on the included series of 10-fold dilutions of the “standard” SPF mouse fecal DNA extract (with the quantified absolute concentration of 16S rRNA gene copies using digital PCR).

Amplification was performed with real-time fluorescence measurements (CFX96 Real-Time PCR Detection System, Bio-Rad Laboratories). Thermocycling conditions were used according to Additional file 1: Table S2. The qPCR data files were analyzed using Bio-Rad CFX Manager 3.1 (#1845000, Bio-Rad Laboratories) and the Cq data were exported to Microsoft Excel for further processing.

### Digital PCR (dPCR) for absolute 16S rRNA gene DNA copy enumeration

Droplet digital PCR (ddPCR) reactions were set up according to [38, 39]. A single replicate reaction volume of 20 μL contained 2.0 μL of the DNA extracts combined with the ddPCR master mix (QX200 ddPCR EvaGreen Supermix, #1864033, Bio-Rad Laboratories), forward and reverse primers (synthesized by Integrated DNA Technologies; Additional file 1: Table S1) at final concentration of 500 nM each, and ultrapure water (Thermo Fisher Scientific).

Droplets were generated using DG8 cartriges (#1864008, Bio-Rad Laboratories), droplet generation oil (#1864006, Bio-Rad Laboratories), and DG8 gaskets (#1863009, Bio-Rad Laboratories) on a QX200 droplet generator (#1864002, Bio-Rad Laboratories) and analyzed using a QX200 Droplet Digital PCR System (#1864001, Bio-Rad Laboratories) using droplet reader oil (#1863004, Bio-Rad Laboratories). The ddPCR data files were analyzed using QuantaSoft Software (#1864011, Bio-Rad Laboratories), and the raw data were exported to Microsoft Excel for further processing.

Thermocycling conditions were used according to [38, 39] and Additional file 1: Table S3. Amplification was performed in PCR plates (#0030133374, Eppendorf, Hauppauge, NY, USA) sealed with pierceable heat seals (#1814040, Bio-Rad Laboratories) using PCR plate sealer (PX1, #1814000, Bio-Rad Laboratories) on a 96-deep well thermocycler (C1000 Touch, #1841100, Bio-Rad Laboratories).

### 16S rRNA gene DNA amplicon barcoding for next-generation sequencing (NGS)

PCR reactions were set up according to [38, 39], in triplicates for each DNA sample. Single-replicate reaction volumes of 30 μL contained 3 μL of the DNA extracts combined with the PCR master mix (5PRIME HotMasterMix, #2200400, Quantabio, Beverly, MA, USA), DNA intercalating dye (EvaGreen, #31000, Biotium, Fremont, CA, USA) at the concentration suggested by the manufacturer (× 1), barcoded forward and reverse primers (synthesized by Integrated DNA Technologies; Additional file 1: Table S1) at a final concentration of 500 nM each, and ultrapure water (Thermo Fisher Scientific). Reactions were set up in 0.2-mL white PCR tubes (#TLS0851) with flat optical caps (#TCS0803, Bio-Rad Laboratories). Thermocycling conditions were used according to [38, 39] and Additional file 1: Table S4. Amplification was performed with real-time fluorescence measurements (CFX96 Real-Time PCR Detection System, Bio-Rad Laboratories) and samples were amplified for a variable number of cycles until the mid-exponential (logarithmic) phase to maximize the amplicon yield and minimize artifacts related to over-amplification [146].

### Digital PCR (dPCR) for Illumina library quantification

Single replicate reaction volume of 20 μL contained 2.0 μL of the diluted amplicon sample ligated with the Illumina adapters, 10 μL of ddPCR master mix (QX200 ddPCR EvaGreen Supermix, #186-4033, Bio-Rad Laboratories), forward and reverse primers (synthesized by Integrated DNA Technologies; Additional file 1: Table S1) targeting the Illumina P5 and P7 adapters respectively at the final concentration of 125 nM each, and ultrapure water (Invitrogen). Thermocycling conditions were used according to Additional file 1: Table S5. PCR amplification and droplet analysis were performed as above.

### Barcoded sample quantification, pooling, library purification, and quality control

Triplicates of each barcoded amplicon sample were combined. Each sample was diluted × 10^5^–10^7^-fold and the molar concentration of barcoded amplicons was quantified using a home-brew ddPCR library quantification assay and KAPA SYBR FAST Universal qPCR Library Quantification Kit (#KK4824, Kapa Biosystems, Wilmington, MA, USA) according to the manufacturer’s instructions (the qPCR reaction was set up same as above).

Barcoded samples were pooled in equimolar amounts. The pooled library was purified using Agencourt AMPure XP beads (#A63880, Beckman Coulter, Brea, CA, USA) according to the manufacturer’s instructions and eluted with ultrapure water (Invitrogen).

The purified library was confirmed to have the 260 nm to 280 nm light absorbance ratio of > 1.8 using a NanoDrop 2000c spectrophotometer (Thermo Fisher Scientific). The average amplicon size of approximately ~ 400 bp was confirmed with a High Sensitivity D1000 ScreenTape System (#5067-5584 and #5067-5585, Agilent Technologies, Santa Clara, CA, USA) using a 2200 TapeStation instrument (Agilent Technologies) and the Agilent 2200 TapeStation Software A.02.01 (Agilent Technologies).

The molar concentration of the pooled library was measured using the ddPCR and KAPA qPCR assays, and the library was submitted for NGS with the sequencing primers described in Additional file 1: Table S1.

### Next-generation sequencing

The library was sequenced on a MiSeq instrument (Illumina, San Diego, CA, USA) in a 300-base-paired-end mode using a MiSeq Reagent Kit v3 (#MS-102-3003, Illumina). PhiX control spike-in was added at 15%.

### PCR primer oligonucleotides (Additional file 1: Table S1)

The same universal microbial 16S rRNA gene V4 primers (modified from [147, 148] and validated in [38, 39] (Barlow JT, Bogatyrev SR, Ismagilov RF: A quantitative sequencing framework for absolute abundance measurements of mucosal and lumenal microbial communities, submitted)) targeting the V4 region of the 16S rRNA gene from the 519 to 806 positions were used for 16S rRNA gene DNA copy quantification and multiplexed microbial community profiling based on 16S rRNA gene amplicon sequencing. Reverse barcoded primers for 16S rRNA gene DNA amplicon barcoding were according to [148].

Primers targeting the P5 and P7 Illumina adapters for barcoded amplicon and pooled library quantification using the ddPCR assay were according to [147–151].

### Sequencing read processing

Demultiplexed 2 × 300 reads were processed using the Qiime2-2019.01 pipeline [152]. DADA2 plugin [153] was used to filter (forward trimming, 5; forward truncation, 230; reverse trimming, 5; reverse truncation, 160), denoise, merge the paired-end sequences, and remove the chimeras. Taxonomic sequence (amplicon sequence variant, ASV) classification was performed using the classifier (available for download from [154]) trained [155] on the V4 515-806 bp regions of 16S rRNA gene sequences from the Silva rRNA reference database, release 132 [156] (available for download from [157]).

Functional gene inference analysis with PICRUSt2 [44, 45] was performed on the ASVs within the Qiime2 environment. Absolute and relative abundances of ASVs were normalized using the inferred 16S rRNA gene DNA copy counts. Obtained predicted metagenome data were used to calculate the normalized relative and absolute abundances of the gene orthologs of interest using Python tools (described below).

### Sequencing data processing

Data handling, calculations, and statistical analyses were performed using Microsoft Excel with the Real Statistics Resource Pack [158], and the Python packages NumPy [159], Pandas [160], SciPy [161], and Statsmodels [162]. Plotting was performed with Matplotlib [163] and Seaborn [164]. All Python packages were run using IPython [165] within Jupyter notebooks [166] distributed with the Anaconda environment [167].

Frequency data for the 16S rRNA gene ASVs assigned to taxa in each sample were converted to relative abundances for each sample. Relative abundances then were converted to absolute abundances using the corresponding values of total 16S rRNA gene DNA loads obtained from the qPCR and ddPCR assays for each sample.

Absolute abundance data were then collapsed to the genus (Fig. 3a) or order (Fig. 3b, c) taxonomical levels using a custom-made Python function (confirmed to yield identical results to the “collapse” method of the Qiime2 “Taxa” plugin [152]). We defined contaminating taxa (from sample handling during collection or from the DNA extraction kit or PCR reagents) using two methods: taxa that were not present in at least 1 out of 16 cecum contents samples (4 mice out of 6 from each group × 4 groups), and taxa identified with a frequency-based contaminant identification [168] implemented by us in Python. Data for chloroplasts and mitochondria of plant origin (likely from the chow diet) were kept in the dataset for Fig. 3a and c and removed for Fig. 3b. Mean absolute abundances of taxa for each group were calculated, converted to relative abundances, and plotted in Fig. 3b.

Principal component analysis (PCA) of the relative abundance data (Additional file 1: Figure S4B) was performed on centered log ratio (CLR)-transformed [169, 170] (after a pseudocount equal to the minimal non-zero sequence count in the dataset was added to all zero values) genus-level relative abundance data using the Python Scikit-learn package [171].

PCA of the absolute abundance data (Fig. 3a) was performed on log_10_-transformed and centered standardized (converted to normally distributed data with mean = 0 and standard deviation = 1) [172] genus-level absolute abundance data using the Python Scikit-learn package [171].

### Bile acid analysis

#### Reagents

TαMCA, TβMCA, TωMCA, THCA, αMCA, βMCA, ωMCA, HCA, HDCA, MCA, GCDCA, GDCA, and GCA (Additional file 1: Table S6) were obtained from Steraloids (Newport, RI, USA).

TCA, CA, DCA, TCDCA, TDCA, TUDCA, TLCA, CDCA, UDCA, LCA, D_4_-TCA, D_4_-DCA, D_4_-CA, D_4_-TDCA, D_4_-GLCA, D_4_-GUDCA, D_4_-GCDCA, D_4_-GCA, and D_4_-GDCA (Additional file 1: Table S6) were obtained from Isosciences (Ambler, PA, USA).

LC/MS grade acetonitrile (#A955-500), water (#W6500), and formic acid (#A117-50) were obtained from Thermo Fisher Scientific.

#### Sample preparation

To overcome sample buffering (pH issues), samples were extracted (using a protocol adapted and modified from [83–85]) in 9 volumes of ethanol with 0.5% formic acid and 9 different heavy isotope (D_4_) internal standards at 5 μM. D_4_ internal standards were taurocholic acid (TCA), cholic acid (CA), deoxycholic acid (DCA), taurodeoxycholic acid (TDCA), glycocholic acid (GCA), glycolithocholic acid (GLCA), glycoursodeoxycholic acid (GUDCA), glycochenodeoxycholic acid (GCDCA), and glycodeoxycholic acid (GDCA). Samples were heated for 1 h at 70 °C with orbital shaking at 900 RPM. Solids were precipitated by centrifugation at 17,000 RCF for 15 min at 4 °C. Supernatants were decanted as 10% of the original sample (e.g., 100 μL of a 1-mL extraction sample) and evaporated at approximately 100 mTorr at RT on a rotovap (Centrivap Concentrator #7810016, Labconco, Kansas City, MO, USA). The evaporated samples were reconsistuted at 100 × dilution from the original sample (e.g., 100 μL decanted solution is resuspended at 1 mL) in 20% acetonitrile, 80% water with 0.1% formic acid.

Due to small volumes, gall bladder bile samples were first diluted in 10 volumes of 100% ethanol (#3916EA, Decon Labs, King of Prussia, PA, USA). The ethanol-based dilutions were combined with 9 volumes of ultrapure water (Invitrogen) and subjected to extraction as above.

Each 10-μL extracted and reconsistuted sample injection was analyzed on a Waters Acquity UPLC coupled to a Xevo-qTOF Mass Spectrometer (Waters, Manchester, UK) using an Acquity UPLC HSS T3 1.8 micron, 2.1 × 10-mm column (# 186003539) and Acquity UPLC HSS T3 1.8 micron Guard Column (# 186003976). Needle wash was two parts isopropanol, one part water, and one part acetonitrile. Purge solvent was 5% acetonitrile in water. A pooled quality control sample was run every eight injections to correct for drift in response.

Mass spectrometer instrument parameters were as follows: Capillary Voltage 2.4 kV, Collision Energy 6.0 eV, Sampling Cone 90 V, Source Offset 40 V, Source 120 °C, desolvation gas temperature 550 °C, cone gas 50 L/h, and desolvation Gas 900 L/h. Time-of-flight mass spectra were collected in resolution mode, corresponding to 30,000 m/Δm. The mass axis was calibrated with sodium formate clusters and locked using leucine enkephalin.

A seven point external calibration curve was collected three times within the run from 0.05 to 30 μM of the bile acid standards (0.05, 0.1, 0.5, 1, 5, 10, and 30 μM). External standards were taurocholic acid (TCA), tauro-alpha-muricholic acid (TαMCA), tauro-beta-muricholic acid (TβMCA), tauro-omega-muricholic acid (TωMCA), tauro-hyocholic acid (THCA), tauro-deoxycholic acid (TDCA), tauro-ursodeoxycholic acid (TUDCA), tauro-chenodeoxycholic acid (TCDCA), taurolithocholic acid (TLCA), glyco-cholic acid (GCA), glyco-hyocholic acid (GHCA), glyco-deoxycholic acid (GDCA), glyco-hyodeoxycholic acid (GHDCA), cholic acid (CA), alpha-muricholic acid (αMCA), beta-muricholic acid (βMCA), omega-muricholic acid (ωMCA), hyocholic acid (HCA, also known as γ-muricholic acid), deoxycholic acid (DCA), chenodeoxycholic acid (CDCA), ursodeoxycholic acid (UDCA), hyodeoxycholic acid (HDCA), murocholic acid (murideoxycholic acid, MDCA), lithocholic acid (LCA), glycolithocholic acid (GLCA), glycourosodeoxycholic acid (GUDCA), and glycochenodeoxycholic acid (GCDCA). It was not possible to resolve UDCA and HDCA; so the sum was reported.

Integrated areas of extracted ion chromatograms were obtained using QuanLynx (Waters, Milford, MA, USA) and a mass extraction window of 10 mDa. Final corrections accounting for drift in instrumental sensitivity were performed in Microsoft Excel.

#### Elution gradient

Samples were eluted using the following gradient of water with 0.1% formic acid (“A”) and balance of acetonitrile with 0.1% formic acid:

1. 0 min, 0.55 mL/min at 68% A
2. 2 min, 0.55 mL/min at 60% A, 10 curve
3. 5 min, 0.55 mL/min at 40% A, 5 curve
4. 6 min, 1.1 mL/min at 0% A, 10 curve
5. 6.2 min, 1.2 mL/min at 0% A, 6 curve
6. 6.5 min, 1.47 mL/min at 0% A, 6 curve
7. 8.9 min, 1.5 mL/min at 0% A, 6 curve
8. 9.0 min, 0.9 mL/min at 68% A, 6 curve
9. 10 min, 0.55 mL/min at 68% A, 6 curve

#### Bile acid data processing

Bile acid data analysis was performed using the tools described in “Sequencing data processing.”

## Supplementary information

**Additional file 1: Figure S1.** Tail cup design and experimental setup for preventing coprophagy. **Figure S2.** Mounting of functional tail cups onto mice. **Figure S3.** Body weight changes across all groups of mice in relation to food intake over the course of the study. **Figure S4.** Quantification of the culturable microbial load and microbiota profile along the entire GIT of mice fitted with functional tail cups (TC-F) and control mice (CTRL). **Figure S5.** Bile acid profiles in gallbladder bile and in lumenal contents along the entire GIT. **Table S1.** Primer oligonucleotide sequences used in the study. **Table S2.** Thermocycling parameters for the quantitative PCR (qPCR) assay for 16S rRNA gene DNA copy quantification. **Table S3.** Thermocycling parameters for the digital PCR (dPCR) assay for absolute 16S rRNA gene DNA copy quantification. **Table S4.** Thermocycling parameters for the 16S rRNA gene DNA amplicon barcoding PCR reaction for next generation sequencing (NGS). **Table S5.** Thermocycling parameters for the digital PCR (dPCR) assay for barcoded amplicon and Illumina NGS library quantification. **Table S6.** Reagents and chemical standards used in the bile acid metabolomics assay. **Table S7.** Bile acid concentrations in gallbladder bile and in lumenal contents along the entire GIT.

## Data Availability

The datasets supporting the conclusions of this article are included within the article and its additional files. Sequencing data (paired end reads in FASTQ) and a manifest file for analysis in Qiime2 are available under a CC-BY license via CaltechDATA: 10.22002/D1.1295. Supplementary Information includes a zip file containing all sequencing sample metadata, numerical microbial quantification data (16S copies from the main study + MPN from the pilot study), Qiime2 sequencing output data, PICRUSt2 output data, numerical bile acid analysis data, numerical body weight data, numerical food intake data, and analytical scripts (iPython Notebooks) for all figures and statistical analyses in the manuscript.
