# Supplementary material for: Self-reinoculation with fecal flora changes microbiota density and composition leading to an altered bile-acid profile in the mouse small intestine
Source: Microbiome. 2020 Feb 12;8:19. doi: 10.1186/s40168-020-0785-4 (PMC7017497; doi:10.1186/s40168-020-0785-4)
Supplement: Supplementary file 2 — Additional file 1: Figure S1. Tail cup design and experimental setup for preventing coprophagy. Figure S2. Mounting of functional tail cups onto mice. Figure S3. Body weight changes across all groups of mice in relation to food intake over the course of the study. Figure S4. Quantification of the culturable microbial load and microbiota profile along the entire GIT of mice fitted with functional tail cups (TC-F) and control mice (CTRL). Figure S5. Bile acid profiles in gallbladder bile and in lumenal contents along the entire GIT. Table S1. Primer oligonucleotide sequences used in the study. Table S2. Thermocycling parameters for the quantitative PCR (qPCR) assay for 16S rRNA gene DNA copy quantification. Table S3. Thermocycling parameters for the digital PCR (dPCR) assay for absolute 16S rRNA gene DNA copy quantification. Table S4. Thermocycling parameters for the 16S rRNA gene DNA amplicon barcoding PCR reaction for next generation sequencing (NGS). Table S5. Thermocycling parameters for the digital PCR (dPCR) assay for barcoded amplicon and Illumina NGS library quantification. Table S6. Reagents and chemical standards used in the bile acid metabolomics assay. Table S7. Bile acid concentrations in gallbladder bile and in lumenal contents along the entire GIT. [file 40168_2020_785_MOESM1_ESM.zip › Supplementary information_Bogatyrev-R.pdf]

Supplementary information for

Self-reinoculation with fecal flora changes microbiota density and composition leading to an altered bile-acid profile in the mouse small intestine

Said Bogatyrev,[1] Justin C. Rolando,[2] and Rustem F. Ismagilov[1,2*]

[1]Division of Biology and Biological Engineering, California Institute of Technology
[2]Division of Chemistry and Chemical Engineering, California Institute of Technology
1200 E. California Blvd., Pasadena, CA, United States of America
* Correspondence to: rustem.admin@caltech.edu

Figures S1-S5

Tables S1-S7

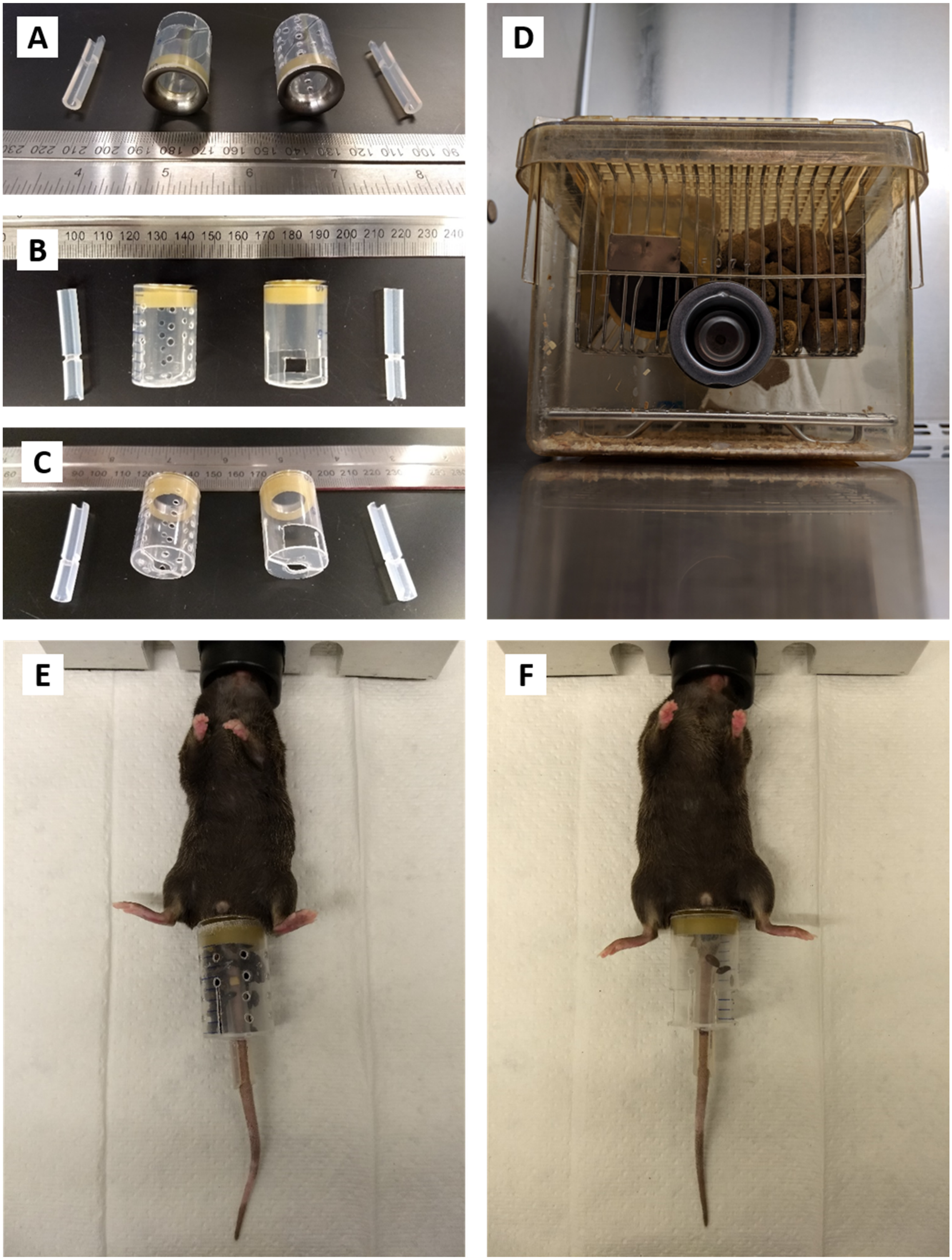

Fig. S1. Tail cup design and experimental setup for preventing coprophagy. (A, B, C) Functional (TC-F, left) and mock (TC-M, right) tail cups as viewed from different perspectives. **(D)** The standard cages with wire mesh floors used in this study (WF). **(E, F)** Ventral view of the functional (TC-F; left) and mock (TC-M, right) tail cups 24 hours after emptying (TC-F) or mock emptying (TC-M).

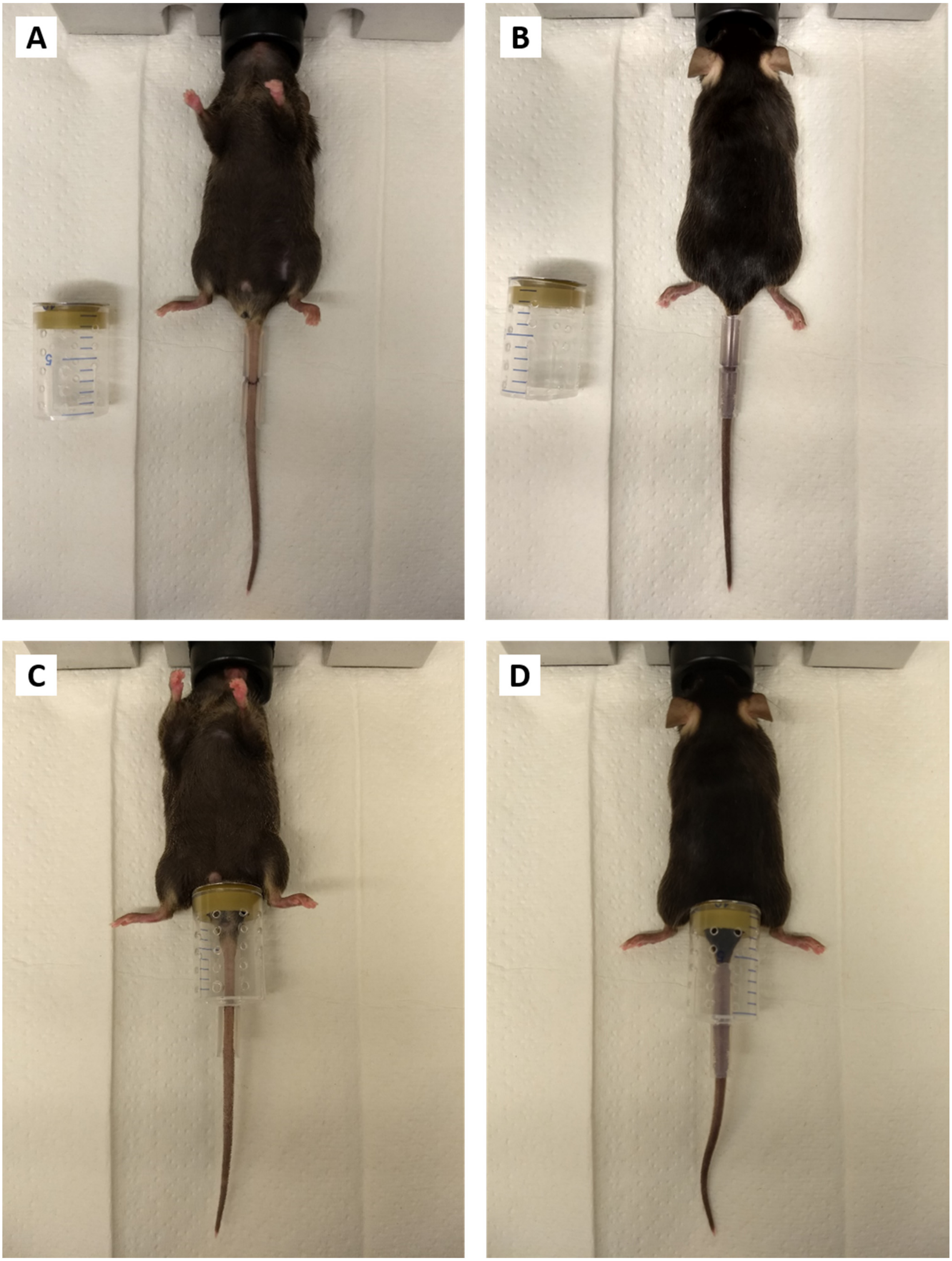

Fig. S2. Mounting of functional tail cups onto mice. (A, B) Ventral and dorsal view of the tail sleeve mounted at the tail base. **(C, D)** Ventral and dorsal view of the functional tail cup installed and locked in place using the tail sleeve.

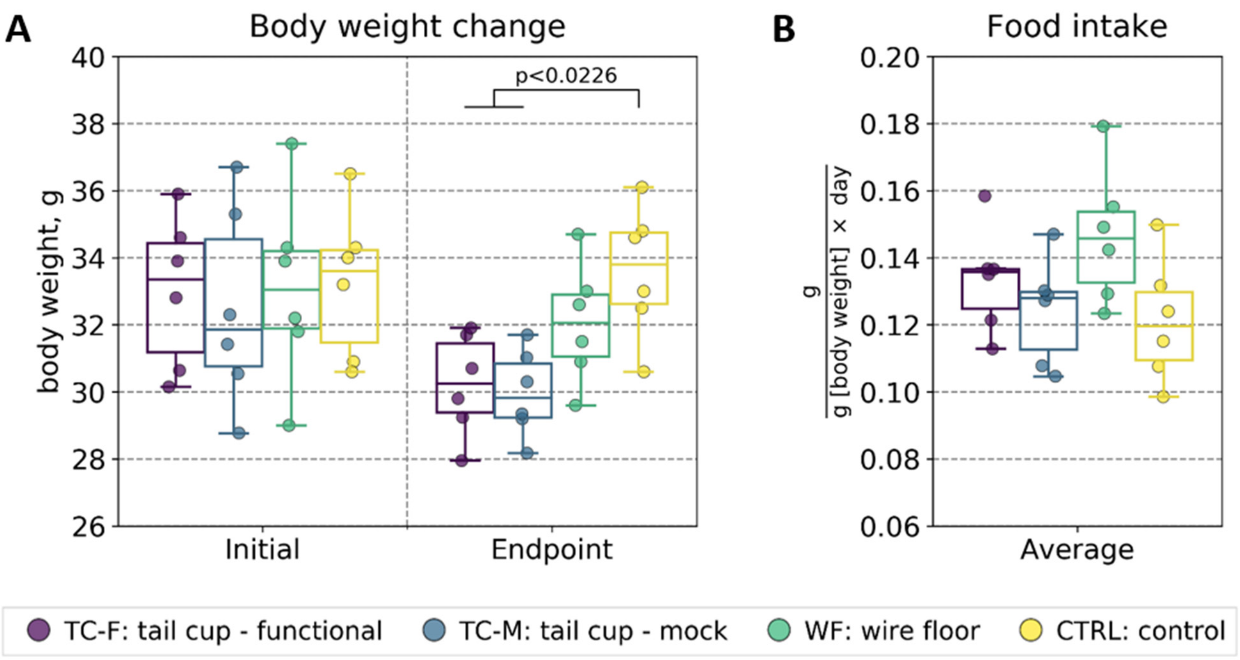

Fig. S3. Body weight changes across all groups of mice in relation to food intake over the course of the study. (A) Body weights of each individual animal at the beginning and at the endpoint of the study. **(B)** Normalized food intake per gram of body weight per day measured over the entire duration of the study. Multiple comparisons of the normally-distributed homoscedastic data were performed using one-way ANOVA; pairwise comparisons were performed using the Student's *t*-test with FDR correction. N = 6 mice per group.

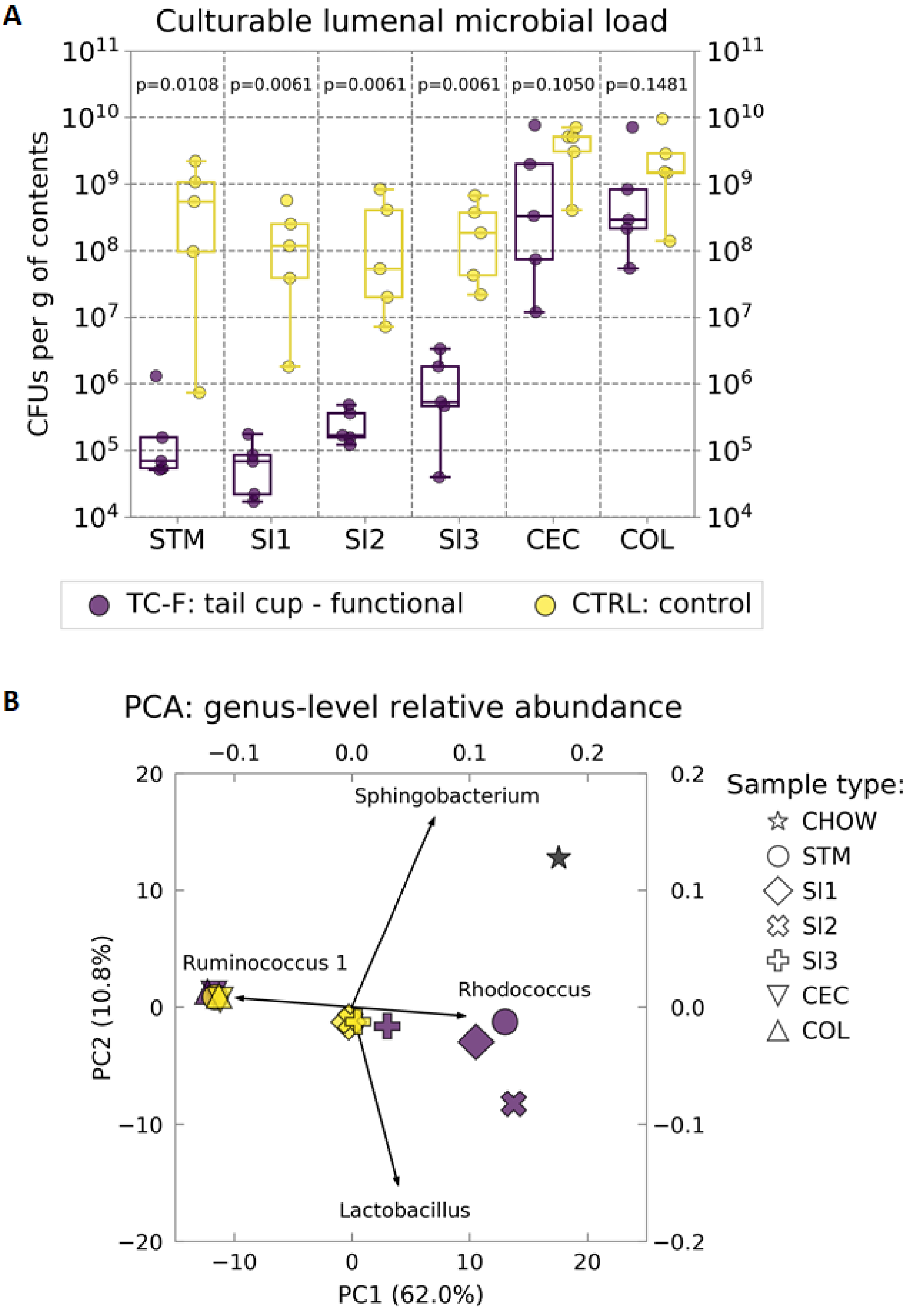

Fig. S4. Quantification of the culturable microbial load and microbiota profile along the entire GIT of mice fitted with functional tail cups (TC-F) and control mice (CTRL). (A) Culturable microbial loads in contents along the gastrointestinal tract were evaluated using the most probable number (MPN) assay performed in anaerobic BHI-S broth (N = 5 mice per group, *P*-values were calculated using the Wilcoxon–Mann–Whitney test). **(B)** PCA analysis of the CLR-transformed relative microbial abundance profiles (16S rRNA gene amplicon sequencing) along the entire GIT in TC and CT mice (N = 1 mouse from each group).

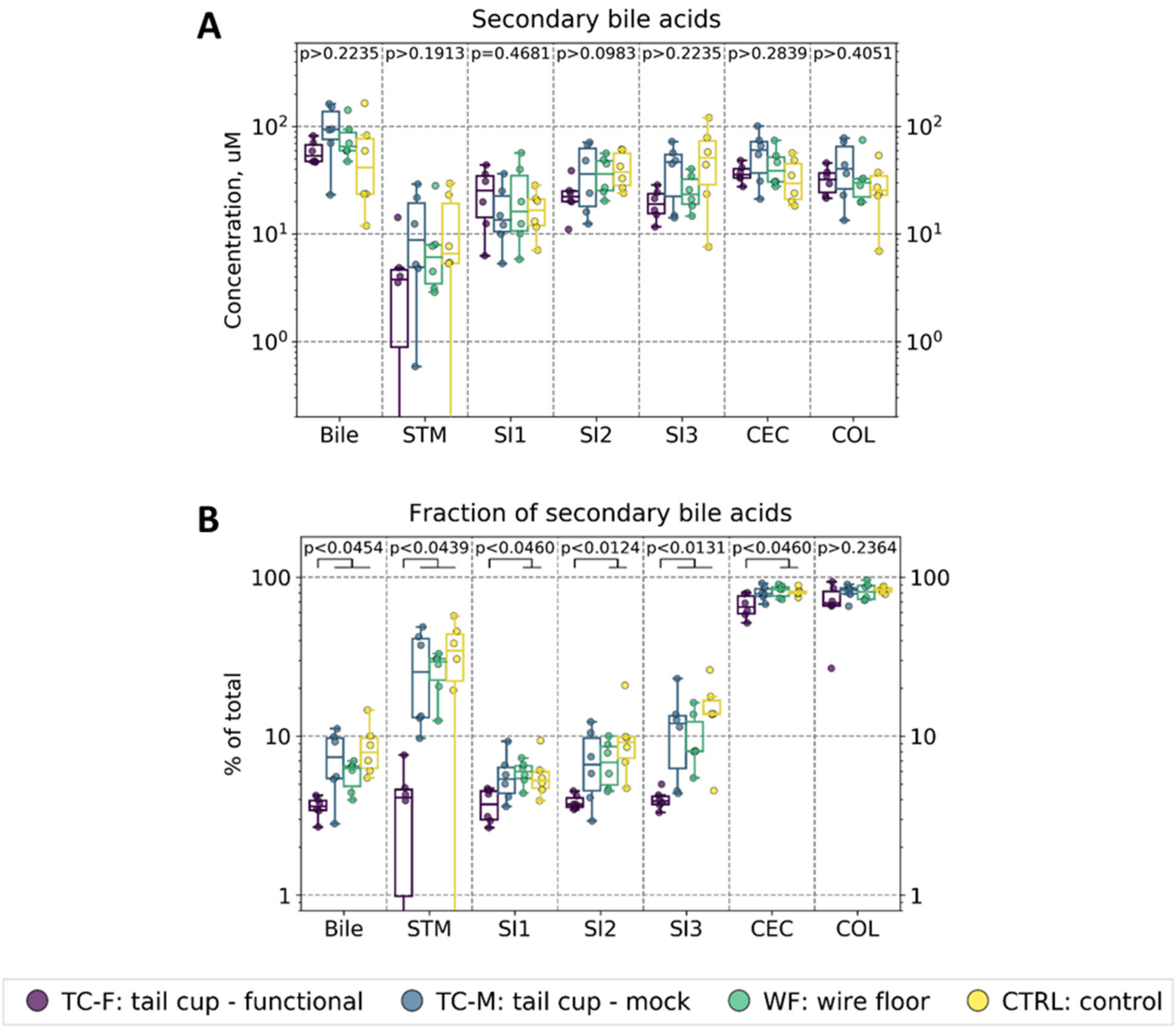

Fig. S5. Bile acid profiles in gallbladder bile and in lumenal contents along the entire GIT. (A) Total secondary bile acid levels (conjugated and unconjugated) and (B) the fraction of secondary bile acids (conjugated + unconjugated) in gallbladder bile and throughout the GIT (STM = stomach; SI1 = upper third of the small intestine (SI), SI2 = middle third or the SI, SI3 = lower third of the SI roughly corresponding to the duodenum, jejunum, and ileum respectively; CEC = cecum; COL = colon). In all plots, individual data points are overlaid onto box-and-whisker plots; whiskers extend from the quartiles (Q2 and Q3) to the last data point within 1.5 × interquartile range (IQR). Multiple comparisons were performed using the Kruskal–Wallis test; pairwise comparisons were performed using the Wilcoxon–Mann–Whitney test with FDR correction. N = 6 mice per group.

Table S1. Primer oligonucleotide sequences used in the study. [NNNNNNNNNNNN] – 12-base barcode sequences "806rcbc" according to [148].

Primer	Oligonucleotide sequence	Assay	Reference
UN00F2	CAGCMGCCGCGGTAA	16S rRNA gene DNA qPCR 16S rRNA gene DNA ddPCR	[38]
UN00R0	GGACTACHVGGGTWTCTAAT		[147, 148]
UN00F2_BC	AATGATACGGCGACCACCGA GATCTACACTATGGTAATTGT CAGCMGCCGCGGTAA	16S rRNA gene DNA amplicon barcoding	[38]
UN00R0_BC	CAAGCAGAAGACGGCATACGAGAT [NNNNNNNNNNNN] AGTCAGTCAGCC GGACTACHVGGGTWTCTAAT		[147, 148]
ILM00F(P5)	AATGATACGGCGACCACCGA	Barcoded amplicon and NGS library quantification ddPCR	[147–151]
ILM00R(P7)	CAAGCAGAAGACGGCATACGA		
Seq_UN00F2_Read_1	TATGGTAATTGTCAGCMGCCGCGGTAA	MiSeq read 1	[38]
Seq_UN00R0_Read_2	AGTCAGTCAGCCGGACTACHVGGGTWTCTAAT	MiSeq read 2	[147, 148]
Seq_UN00R0_RC_Index	ATTAGAWACCCBDGTAGTCCGGCTGACTGACT	MiSeq index read	[147, 148]

Table S2. Thermocycling parameters for the quantitative PCR (qPCR) assay for 16S rRNA gene DNA copy quantification.

Step	Repeats	Temperature, °C	Time, sec
Initial denaturation	× 1	95	120
Cycle	× 40	95	15
		53-54	10
		68	45

Table S3. Thermocycling parameters for the digital PCR (dPCR) assay for absolute 16S rRNA gene DNA copy quantification.

Step	Repeats	Temperature, °C	Time, sec	Ramp, °C/sec
Initial denaturation	× 1	95	300	2.0
Cycle	× 40	95	30	2.0
		52	30	2.0
		68	60	2.0
Dye stabilization	× 1	4	300	2.0
		90	300	2.0
		12	∞	2.0

Table S4. Thermocycling parameters for the 16S rRNA gene DNA amplicon barcoding PCR reaction for next generation sequencing (NGS).

Step	Repeats	Temperature, °C	Time, sec
Initial denaturation	× 1	94	180
Cycle	× var.	94	45
		54	60
		72	105
Final extension	× 1	72	600

Table S5. Thermocycling parameters for the digital PCR (dPCR) assay for barcoded amplicon and Illumina NGS library quantification.

Step	Repeats	Temperature, °C	Time, sec	Ramp, °C/sec
Initial denaturation	× 1	95	300	2.0
Cycle	× 40	95	30	2.0

		60	90	2.0
Dye stabilization	× 1	4	300	2.0
		90	300	2.0
		12	∞	2.0

Table S6. Reagents and chemical standards used in the bile acid metabolomics assay.

Bile acid	Reference #	Vendor	LOT
TαMCA	C1893-000	Steraloids	B1439
TβMCA	C1899-000	Steraloids	B1594
TωMCA	C1889-000	Steraloids	B1731
THCA	C1887-000	Steraloids	B1621
αMCA	C1890-000	Steraloids	B1529
βMCA	C1895-000	Steraloids	B1725
ωMCA	C1888-000	Steraloids	B1710
HCA (gMCA)	C1850-000	Steraloids	B0696
HDCA	C0860-000	Steraloids	B0684
MCA	C0910-000	Steraloids	B1711
GDCA	C1087-000	Steraloids	B2122
GCA	C1927-000	Steraloids	
GHDCA	C0865-000	Steraloids	B1667
GHCA	C1860-000	Steraloids	L1105
TCA	13232UNL	Isosciences	EH1-2015-111A1
CA	13098UNL	Isosciences	EH1-2014-075A1
DCA	13100UNL	Isosciences	EH1-2014-076A1
TCDCA	13105UNL	Isosciences	EH1-2015-110A1
TDCA	13225UNL	Isosciences	EH1-2015-112A1
TUDCA	13106UNL	Isosciences	EH1-2014-027A1
TLCA	13230UNL	Isosciences	EH1-2014-077A1
CDCA	13101UNL	Isosciences	PG1-2014-149A1
UDCA	13102UNL	Isosciences	EH1-2015-113A1
LCA	13099UNL	Isosciences	EH1-2014-030A1
D4-TCA	13232	Isosciences	SJ5-2015-035A1
D4-DCA	13100	Isosciences	RS6-2014-168A1
D4-CA	13098	Isosciences	SJ5-2015-100A1
D4-TDCA	13225	Isosciences	SJ5-2015-034A1
D4-GLCA	13231	Isosciences	SR3-2015-203A1
D4-GUDCA	13224	Isosciences	SJ5-2017-206A1

D4-GCDCA	13104	Isosciences	SJ4-2012-070A1
D4-GCA	13443	Isosciences	SJ5-2015-118A1
D4-GDCA	13226	Isosciences	SJ5-2015-033A1

Legend for Table S7 (attached as .csv file):

Table S7. Bile acid concentrations in gallbladder bile and in lumenal contents along the entire GIT.
